# Anti-inflammatory activities of *Coleus forsteri* (formerly *Plectranthus forsteri*) extracts on human macrophages and chemical characterization

**DOI:** 10.3389/fphar.2022.1081310

**Published:** 2023-01-09

**Authors:** Mael Nicolas, Malia Lasalo, Sharron Chow, Cyril Antheaume, Karl Huet, Edouard Hnawia, Gilles J. Guillemin, Mohammed Nour, Mariko Matsui

**Affiliations:** ^1^ Département de Chimie, Université Côte d’Azur, Nice, France; ^2^ Group Bioactivities of Natural compounds and derivatives (BIONA), Formerly Group Immunity and Inflammation (GIMIN), Institut Pasteur of New Caledonia, Member of the Pasteur Network, Noumea, New Caledonia; ^3^ Neuroinflammation Group, Macquarie Medical School, Macquarie University, Sydney, NSW, Australia; ^4^ Institut de Science et d’Ingénierie Supramoléculaires, Université de Strasbourg, Strasbourg, France; ^5^ PHARMADEV, UMR152, Institut de Recherche pour le Développement (IRD), Noumea Center, Noumea, New Caledonia; ^6^ Institut des Sciences Exactes et Appliqués (ISEA), EA7484, Université de Nouvelle-Calédonie, Noumea, New Caledonia

**Keywords:** *Coleus forsteri*, traditional plant, anti-inflammatory, macrophage, cytokine, quinolinic acid (QUIN), abietane, diterpene

## Abstract

**Introduction:** Formerly named *Plectranthus* *forsteri*, *Coleus forsteri* (Benth.) A.J.Paton, 2019 is a Lamiaceae traditionally used to treat flu-like symptoms and shock-related ecchymosis, especially in the Pacific region. Few studies investigated chemical composition and anti-inflammatory potential of this plant.

**Method:** Herein, we investigated anti-inflammatory potential of *C. forsteri* ethanolic (ePE) and cyclohexane (cPE) plant extract on LPS-induced human macrophages models and quantified cytokines and quinolinic acid (QUIN) as inflammatory markers.

**Results:** Our results show that extract of ePE and cPE significantly inhibit inflammatory cytokine IL-6 and TNF-α induced by LPS on PMA-derived THP-1 macrophages. QUIN production is also diminished under ePE and cPE treatment in activated human monocyte-derived macrophages (MDMs). Seven abietane diterpenes were characterized from *C. forsteri* cPE including coleon U (**1**), coleon U-quinone (**2**), 8α,9α-epoxycoleon U-quinone (**3**), horminone or 7α-hydroxyroyleanone (**4**), 6β,7α-dihydroxyroyleanone (**5**), 7α-acetoxy-6β-hydroxyroyleanone (**6**) and 7α-formyloxy-6β-hydroxyroyleanone (**7**).

**Discussion:** We discussed potential contributions of these molecules from *C. forsteri* extracts for their anti-inflammatory activities.

## 1 Introduction

Immune-mediated inflammatory diseases (IMIDs) represent a group of diseases commonly sharing immune dysregulation (Kuek et al., 2007; El-Gabalawy et al., 2010). IMIDs include rheumatoid arthritis (RA), the spondyloarthritis (SpA) disease spectrum, systemic lupus erythematosus (SLE), connective tissue disorders, cutaneous inflammatory conditions like psoriasis and atopic dermatitis, inflammatory bowel disease (IBD), Crohn’s disease (CD) and ulcerative colitis (UC), asthma and autoimmune neurological diseases such as multiple sclerosis. The prevalence of IMIDs varies depending on the populations, and the overall prevalence of IMIDs in the Western society was estimated between 5% and 7% in 2010 (El-Gabalawy et al., 2010). More recently, 362,150 deaths were attributed to the top 15 IMIDs from 2013 to 2017 in the U.S. (Singh et al., 2020). The prevalence of IBD globally increased from 1990 to 2017 to reach 6.8 million cases worldwide in 2017 (Alatab et al., 2020). IMIDS has been considered for a long time to affect populations mainly from developed countries. Recent epidemiological studies showed that IMIDs is becoming increasingly common among immigrants from developing countries with accelerating incidence of IBD in recently industrialized regions (Ng et al., 2017; Agrawal et al., 2019; Quaresma et al., 2022). IMIDs are characterized by acute or chronic inflammation that can affect any organ and biological systems, leading to morbidity, reduced quality of life and premature death (El-Gabalawy et al., 2010).

Cytokines are critical mediators of the inflammatory process. They are produced to trigger the host immune response during infectious diseases and are also major drivers of the pathogenic inflammatory response in IMIDs. Exacerbated cytokine production translate as an amplification of the inflammatory responses leading to multiple deleterious biological effects. Treatments with corticoids and non-steroidal anti-inflammatory drugs (NSAIDs) are commonly prescribed to regulate the inflammatory response in IMIDs but long term therapy are associated with severe side effects, such as gastrointestinal ulceration and bleeding, osteoporosis, hypertension and glaucoma (Gautam and Jachak, 2009; Kondo and Amano, 2018; Wongrakpanich et al., 2018). The development of new therapies is targeting signaling pathways linked with inflammatory cytokines such as tumor necrosis factor (TNF), interleukins (ILs) or interferons (IFNs) mainly with the use of monoclonal antibodies (Kuek et al., 2007; Kondo and Amano, 2018; McInnes and Gravallese, 2021). Although these therapies show some clinical efficacy, patients might be or might become refractory to monoclonal antibody treatment as observed in 20%–40% of IBD-suffering patients. These type of therapies can also be associated with the development of opportunistic diseases related to immunosuppressive mechanisms (Hoffmann et al., 2017; Baker and Isaacs, 2018). These targeted treatments are also used to limit acute inflammation; “cytokine storm”, in patients with sepsis or COVID-19 (Athale et al., 2022) with variable success (Huang et al., 2019).

The kynurenine pathway (KP) is a major catabolic pathway of the essential amino acid tryptophan (TRP). The enzyme 2,3-dioxygenase (IDO1) is activated in the KP by inflammation molecules leading to the production of several bioactive metabolites such as kynurenine (KYN) and quinolinic acid (QUIN). QUIN induces the production of free radicals and exerts neuronal excitotoxicity resulting in neuronal damage and death. Activated human macrophages and peripheral monocytes showed a significant elevation of KP enzyme expression and metabolite production (Guillemin et al., 2003a; Guillemin et al., 2003b; Guillemin et al., 2003c; Jones et al., 2015). Dysregulation of the KP and abnormal levels of the KP metabolites were reported in autoimmune neurological diseases such as multiple sclerosis and contribute to their pathogenesis (Lovelace et al., 2016; Pires et al., 2022; Sundaram et al., 2022). Several clinical trials are currently assessing inhibitors of the KP targeting the IDO1 enzyme (Modoux et al., 2021; Pires et al., 2022). Natural products (NPs) are also explored as potential IDO1 inhibitors (Chen et al., 2012; Bhat et al., 2021).

**FIGURE 1:**
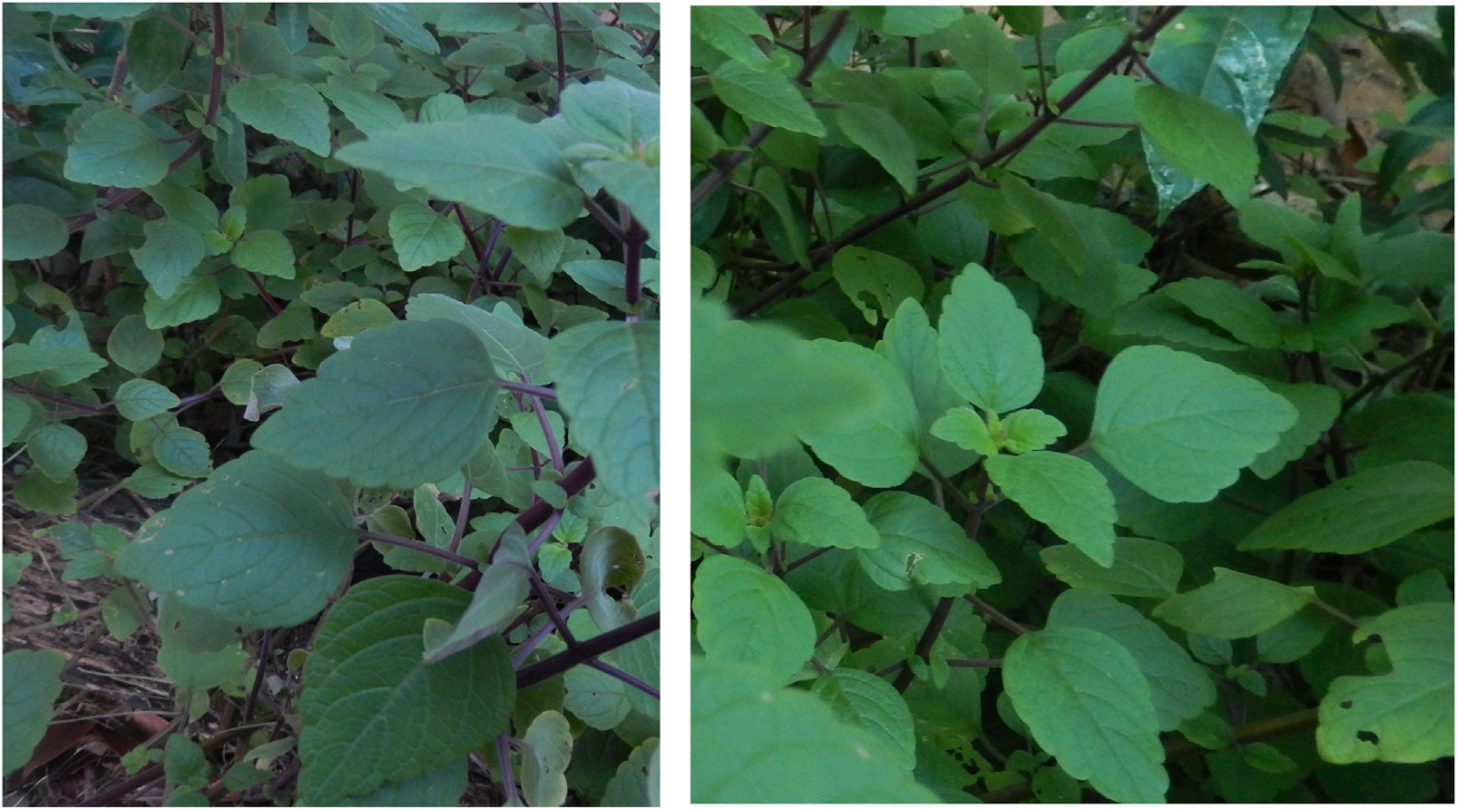
*Coleus forsteri* (Benth.) A.J. Paton (2019). Photos by Dr Edouard Hnawia.

Multiple NPs including plant extracts from traditional medicines are known to have significant anti-inflammatory effects by regulating of cytokine production and can be used for several inflammatory diseases such as RA, atopic dermatitis and IBD (Ghasemian et al., 2016; Luo et al., 2020; Wu et al., 2021; Zeng et al., 2022). Although the traditional knowledge about medicinal NPs is progressively getting lost, traditional plants are still commonly used in the Pacific region (Rageau and Schmid, 1973; Bourdy et al., 1992; Whistler, 1992). Among those plants, the large and medicinally important tropical plant genus *Plectranthus sensu lato* (Lamiaceae, subfamily Nepetoideae) was recently split into three separate genera based on molecular and morphological evidence: *Plectranthus sensu stricto* (72 species), *Coleus* (294 species) and *Equilabium* (42 species) (Paton et al., 2018; Paton et al., 2019). Previously classified as a *Plectranthus* species, *Coleus forsteri* (Benth.) A.J.Paton, 2019 (Figure 1) is an herbaceous plant found in the Southwest Pacific especially in New Caledonia, Vanuatu, Fiji Island, Tonga (Rageau and Schmid, 1973; Lormée et al., 2011; Paton et al., 2019). Named “Hmitre” or “Hnizi” or “Arnica kanak” by New Caledonian traditional peoples, the whole plant or leave extracts of *C. forsteri* are traditionally used to treat flu symptoms or as analgesic against shock (Rageau and Schmid, 1973; Suprin, 2008; Lormée et al., 2011). To our knowledge, only few chemical studies characterized polyphenols and diterpenoids from *C. forsteri* methanolic extract and essential oil, but no anti-inflammatory bioactivity has been published (Grayer et al., 2003; Wellsow et al., 2006; Kubínová et al., 2013). Herein, we assessed the anti-inflammatory potential and phytochemical profile of *C. forsteri* whole plant extracts. For this purpose, we studied the effect of the plant extracts on cytokine and QUIN production using lipopolysaccharide (LPS)-induced human macrophage *in vitro* models. Molecules from the plant cyclohexane extract were also characterized.

## 2 Materials and methods

### 2.1 Chemicals and reagents

All chemicals and reagents were obtained from Sigma (St Louis, United States), unless otherwise stated.

### 2.2 Plant collection and identification

*C. fosteri* whole plants were manually collected in Noumea, South Province, New Caledonia (S22°16', E166°27'). Authorization for sampling was accorded by the South Province (N°20798-2017/ARR/DENV du 25/09/2017). Specimen sample was referenced (NL189F2/QMC3) and conserved by the ISEA unit at the University of New Caledonia (UNC). Species identification was confirmed by botanists from the French National Research for Sustainable Development, New Caledonia IRD Center.

### 2.3 Preparation of plant extract

Whole plants were dried at 37 °C for 2 weeks and grounded. Dried powder (60 g) was extracted twice in 600 ml of cyclohexane for 4 h with ultrasonic treatment followed by two extractions with 600 ml of EtOH/H_2_O (70:30) for 4 h with ultrasonic treatment. Cyclohexane and hydroalcoholic extracts were filtered on Büchner and solvents evaporated to obtain 1.8 g and 7.9 g of dried extracts respectively, corresponding to 3.0% and 13.1% extraction yields. These cyclohexane (cPE) and ethanol (ePE) extracts of *C. fosteri* whole plant were referenced (QMC3 cyclo and QMC3 EtOH respectively) and conserved at −20°C until proceeding to biological assays.

### 2.4 Cell cultures and differentiation

The human THP-1 cell line (ATCC^®^ TIB-202™) and the monocyte-derived macrophage (MDM) primary culture were used as *in vitro* models to study the anti-inflammatory effect of *C. forsteri* extracts. Cultures were maintained at 37°C in humidified atmosphere at 5% CO_2_ in specific culture media described below. THP-1 cells were cultured in RPMI 1640 medium (Corning; 10-043-CV) supplemented with HEPES 1M (Pan Biotech; P05-01100), 10% Fetal Bovine Serum (Dominique Dutscher; S 181B-500), 1% L-glutamine (Gibco; 35050-061) and 1% antibiotic (Antibiotic-Antimycotic 100X, Gibco; 15240-062). Monocytes were counted and 5 × 10^6^ cells were resuspended at each passage once a week. Cells were seeded in 24-wells cell culture plates (1 × 10^6^ cells/well, NEST, 0999052) and differentiated into macrophages after treatment with Phorbol 12-myristate 13-acetate (PMA, Sigma-Aldrich; P8139) at 10 ng/ml for 48 h (Schwende et al., 1996; Leu et al., 2019). Monocyte-derived macrophages (MDMs) were obtained from human blood monocytes purified from peripheral blood mononuclear cells (PBMCs). Briefly, PBMCs were isolated from healthy buffy coats obtained from the Australian Red Cross blood service under material supply agreement 17-07NSW-06. Buffy coats were diluted at equal volume of phosphate-buffered saline (PBS) and layered over a density gradient Lymphoprep™ (STEMCELL Technologies; 07861) and centrifuged at 400 × g for 40 min at 20°C with no brake. The isolated PBMC layer was collected and washed with PBS twice at 100 × g for 10 min at 20°C with brake. Cell pellets were resuspended in RPMI-1640 (ThermoFisher Scientific; 11875093) with 10% human serum (Australian Red Cross), 1% antibiotic-antimycotic (ThermoFisher Scientific; 15240062) and seeded in 24-well plate (∼0.25 × 106 cells/well) and left to recover overnight at 37°C with 5% CO2 in a humidified atmosphere. Next day, the cells were washed twice with PBS. The adherent monocytes were cultured in complete RPMI with recombinant human (rh) GM-CSF (50 ng/ml) (BioLegend; 572903) for 6 days to allow macrophage differentiation (Manéglier et al., 2009; Gray et al., 2013).

### 2.5 Induction and treatments

PMA-differentiated THP-1 macrophages were stimulated with LPS from *E. coli* 0111:B4 (Sigma-Aldrich; L2630) at 1 μg/ml with or without exposure to plant extracts at the final concentration of 25 μg/ml in DMSO 0.025%. Controls include dexamethasone (Sigma–Aldrich; D4902-100 MG) at 127.5 nM and cells without treatment. Following 24 h of induction, supernatants were centrifuged at 1,200 rpm for 7 min at 4°C and one part was used for cytotoxicity assay while the other was conserved at −20°C until cytokines quantification. Experiments were performed three times with 3 replicates for each condition. MDMs were washed twice with PBS to remove rhGM-CSF and then induced with LPS from *E. coli* O55:B5 (Sigma-Aldrich; L6529-1 MG) at 20 ng/ml during 48 h in presence or not of plant extracts at the final concentrations of 10 μg/ml, 25 μg/ml or 50 μg/ml in DMSO 0.05%. Experiments were reproduced three times with at least 2 replicates for each condition. After treatments, supernatants were collected and kept at −20°C for QUIN quantification.

### 2.6 Determination of cell mortality

Cytotoxicity was evaluated by quantifying the release of lactate dehydrogenase (LDH) in the culture supernatant that correlates with the amount of cell death and membrane damage, providing an accurate measurement of cellular toxicity (Haslam et al., 2000). For THP-1, LDH was quantified using the commercial CytoTox 96^®^ Non-Radioactive Cytotoxicity Assay (Promega; G1780) following the manufacturer’s specifications. Absorbance at 450 nm (A_450_) was read using a microplate spectrophotometer (Multiskan™ FC, Thermo Fisher Scientific). LDH in the supernatants was normalized against absorbance obtained for total lysed cells, and results were expressed as percent of cytotoxicity (Sinyeue et al., 2022). For MDMs, LDH was quantified using the commercial LDH-Glo™ Cytotoxicity Assay (Promega; J2381) following the manufacturer’s specifications. The luminescent signal was read, using a luminescence mode of BMG CLARIOstar Plus plate reader. LDH in the supernatants of treatments was normalized against that obtained from negative control and results were expressed as percent of cytotoxicity.

### 2.7 Quantification of cytokines by ELISA

Cytokines production in supernatants was quantified according to the manufacturer’s instructions for human TNF-α, human IL-6, human IL-10 and human IL-1β (DuoSet, R&D Systems). Absorbance was measured at 570 nm and 450 nm on the microplate spectrophotometer (Multiskan ™ FC, Thermo Fisher Scientific). Afterward, the protein concentrations were calculating using the absorbance based on the regression equation with each cytokine standard and expressed as picograms per milliliter (pg/ml).

### 2.8 Quantification of QUIN by gas chromatography/mass spectrometry (GC/MS)

GC/MS was used to quantify QUIN concentrations in cell culture supernatants as previously described (Smythe et al., 2002; Guillemin et al., 2003b; Guillemin et al., 2007). 100 µL of the deproteinized sample were derivatized for quantification of QUIN using an Agilent 7890A GC system coupled with Agilent 5975C mass spectrometry detector and Agilent 7693 A autosampler (Agilent Technologies Inc., Santa Clara, CA). The separation of QUIN was carried out with a DB-5MS column, 0.25 µm film thickness, 0.25 mm × 30 m capillary column (Agilent Technologies Inc., Santa Clara, CA) for 12 min. Concentrations of QUIN were analyzed using Agilent GC/MSD ChemStation software (Edition 02.02.1431). A series of deuterated and non-deuterated standards for QUIN were used for a six-point calibration curve to interpolate the quantity of the sample readout.

### 2.9 Chemical fractionation

The cPE extract was first analyzed by HPLC/MS and the major compounds present in this fraction isolated by semi preparative HPLC to yield compounds (1) to (7). Dried extracts were solubilized in methanol at 10 mg/ml for analytical and semi preparative experiments, the injected volume being different, and filtered on a 0.45 μM PTFE filter before HPLC analysis. The cPE samples were analyzed by HPLC-UV-DAD (Waters 2695) at different wavelengths, using a RP Nucleodur C18ec column (250 by 4.6 mm, 5 μm particle size, Macherey Nagel) column and using water plus formic acid 0.05% as solvent A, and acetonitrile as solvent B plus formic acid 0.05%, and coupled with mass spectrometry (MS) using an ion trap Bruker Esquire HCT Ultra mass spectrometry instrument equipped with an electrospray ion source in positive and negative mode (data were viewed by using Hystar Bruker software). The analytical conditions were optimized to enhance the separation of the compounds present in this fraction. An isocratic method (solvent A: 20%, solvent 80B: %, flow rate: 1.5 ml min^−1^) was chosen. HPLC quality solvents were purchased from Fischer Chemicals (Leicestershire, United Kingdom).

### 2.10 Structural elucidation

Chemical structures were determined by 1D NMR ^1^H (600 MHz), ^13^C (150 MHz) and 2D homo using the NMR equipment from Brucker with the following parameters: 600 MHz Avance III, equipped with a 5 mm BBFO + probe, and hetero-coupling methods (COSY, HSQCed, HMBC and NOESY) in CDCl_3_ at 298 K and data were compared to those found in the literature. The numeration for the carbon atoms is shown in Figure 4 for coleon U (1) and is the classical one used for abietane diterpenes. The results obtained for ^1^H and ^13^C NMR are gathered in tables 2 and 3. δ in ppm (s: singlet, d: doublet, t: triplet, q: quadruplet, quint: quintuplet, sept: septuplet, m: multiplet, l: large, J in Hz, number of protons). Mass spectra were in accordance with the structures found (Table 1).

**TABLE 1:** Mass Spectrometry data for compounds (1) to (7) isolated from *C. forsteri* cyclohexane extract.

Peaks	tr (min)	UV (max)	Mass spectrometry (mode)	Formula	M u.m. (exact mass)	MS(m/z)				
						[M+H]^+^	[M+Na]^+^	[2M+Na]^+^	[M-H]^−^	
peak 1 **(5)**	28,4	270	esi + and -	C20H28O5	348.19	349.4	371.4	719.5	347.2	loss 2 times 18 (H2O/OH)
peak 2 **(3)**	29.3	310	esi + and -	C20H24O6	360.15	361.3	383.3	743.5	359.2	
peak 3 **(2)**	29,5	245, 450	esi + and -	C20H24O5	344.16	345.3	367.3	711.5	342.2	
peak 4 **(7)**	29,8	270	esi + and -	C21H28O6	376.18	*	399.4	775.6	375.2	M + H-44 (-COO)
peak 6 **(6)**	32	275	esi + and -	C22H30O6	390.2	391.4	*	*	389.2	loss 44 (-COO) and 18 (H2O/OH)
peak 7 **(4)**	32,6	280	esi-	C20H28O4	332.19	*	*	*	331.2	
peak 9 **(1)**	33,2	288, 380	esi + and -	C20H26O5	346.17	347.3	*	*	345.2	loss 18 (H2O/OH) and 28 (CO)

### 2.11 Statistical analyses

Results are provided as mean ± standard deviation (SD). Statistical analyses were performed using the software statistical package Prism 9.0 (GraphPad Software LLC, United States). Cytotoxicity and inhibitory effect on QUIN production were evaluated using a Kruskal-Wallis test followed by a Dunn’s multiple comparison test. Inhibitory response on cytokine production was evaluated using a Mann–Whitney non-parametric test to compare distribution between treatments and LPS induction. *p* values <0.05 were considered significant.

## 3 Results

### 3.1 Anti-inflammatory potential of *C. forsteri* extracts on LPS-induced cytokines in human THP-1 macrophages

**FIGURE 2:**
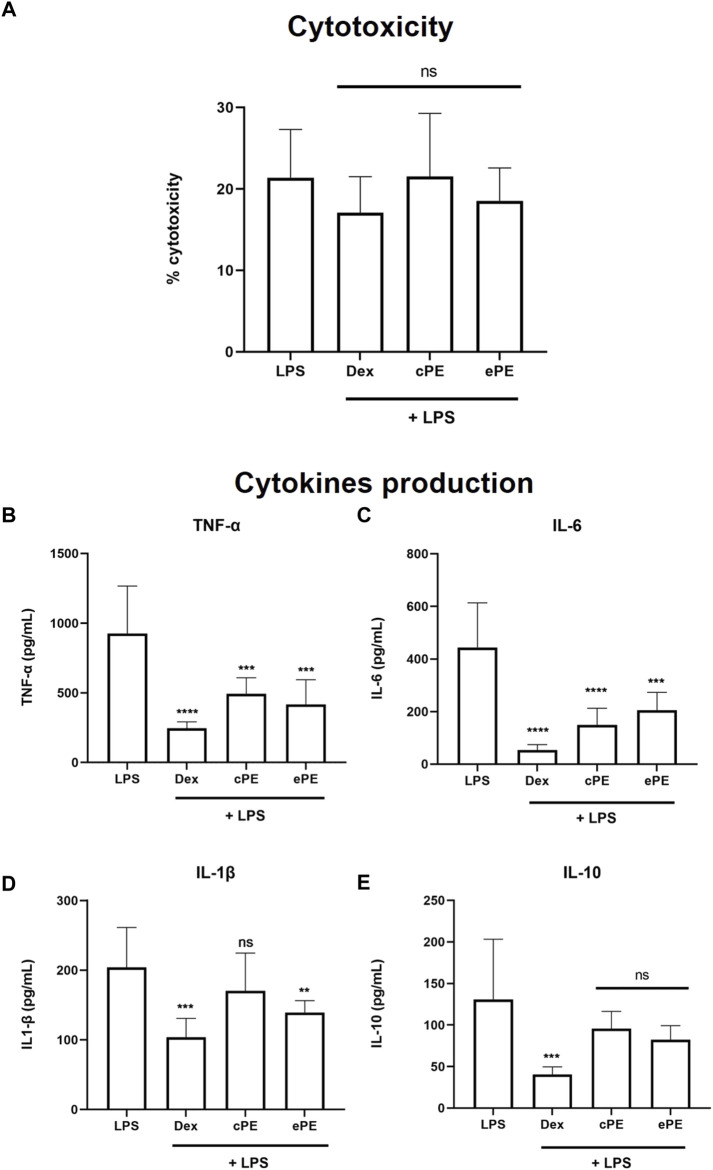
Inhibitory effect of *C. forsteri* extracts on cytokine production. Anti-inflammatory potential of *C. forsteri* cyclohexane (cPE) and ethanolic (ePE) plant extract at 25 μg/ml was evaluated on cytokines produced by PMA-THP-1 macrophages induced with LPS (1 μg/ml) for 24 h. Dexamethasone (DEX) at 127 nM was used as positive inhibitory control. Cytotoxicity of cPE and ePE was evaluated on LPS-induced PMA-THP-1 macrophages **(A)** as described in Materials and Methods. Cytokines IL-1β **(B)**, IL-6 **(C)**, IL-10 **(D)** and TNF-α **(E)** were quantified using ELISA technique. Mann-Whitney analyses were used for comparison between treatments compared to control or LPS condition. *, *p* < 0.05; **, *p* < 0.01; ***, *p* < 0.005; ****, *p* < 0.001; ns, non-significant.

To evaluate potential plant extract cytotoxicity on THP-1 cells, LDH was quantified after incubation with plant extracts at 25 μg/ml. No significant increase of cytotoxicity was observed between the LPS-induced cells with or without plant extract treatment, with cytotoxicity ranging from 18.53 ± 4.029% to 21.35 ± 5.938% (Figure 2A). Thus, concentration at 25 μg/ml for plant extract was confirmed for further studies. Incubation with cPE and ePE at 25 μg/ml decreased the production of LPS-induced TNF-α from 925.5 ± 341.1 pg/ml in LPS condition to 493.8 ± 113.9 and 417.6 ± 176.4 pg/ml when treated with cPE and ePE, respectively (Figure 2B). Similarly, plant extracts also decrease LPS-dependent IL-6 production (Figure 2C) compared to the LPS-treated cells. Results showed that plant extracts have different effects on IL-1β cytokine production depending on ethanolic or cyclohexane extracts. Only the ePE significantly reduced the production of IL-1β to 139.3 ± 16.99 pg/ml compared IL-1β level at 203.9 ± 57.45 pg/ml for LPS-treated cells (Figure 2D). In contrast, there was no significant effect on the production of LPS-dependent IL-10 production regardless the exposure of plant extract, with a concentration of cytokine ranging from 82.12 ± 17.09 to 130.6 ± 72.58 pg/ml (Figure 2E).

### 3.2 Inhibitory effect of *C. forsteri* extracts on LPS-dependent quinolinic acid production in human primary MDMs

**FIGURE 3:**
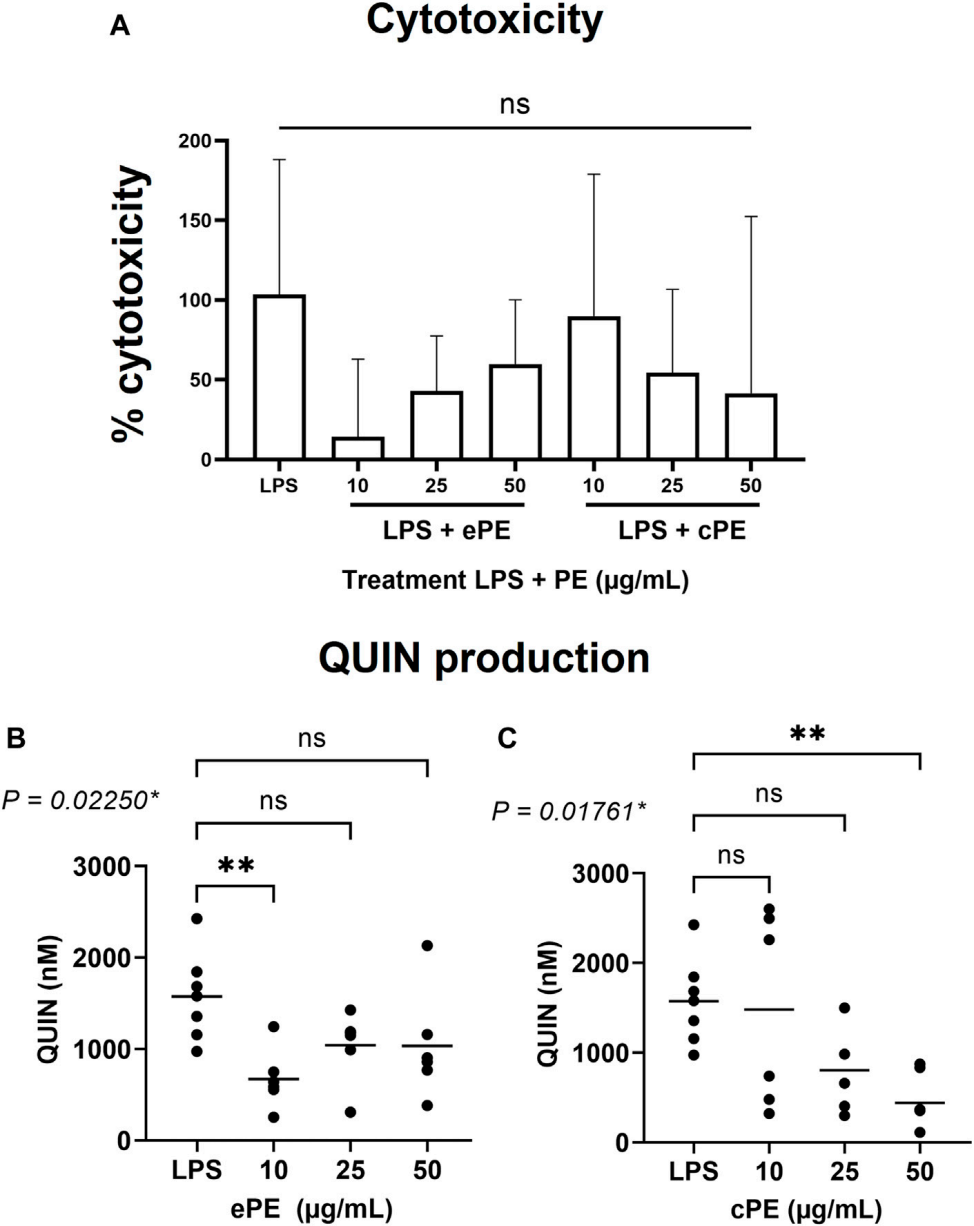
Inhibitory effect of *C. forsteri* extracts on QUIN production. Anti-inflammatory potential of *C. forsteri* cyclohexane (cPE) and ethanolic (ePE) plant extract at 10, 25 or 50 μg/ml were evaluated on QUIN produced by MDMs induced with LPS (20 ng/ml) for 48H. Cytotoxicity of cPE and ePE was evaluated on MDMs **(A)** as described in Materials and Methods. QUIN was quantified by GC/MS after treatment with ePE **(B)** and cPE **(C)** at various concentrations. Kruskal-Wallis test followed by a Dunn’s multiple comparison test were used for comparison between treatments compared to LPS condition. *, *p* < 0.05; **, *p* < 0.01; ns, non-significant.

Cytotoxicity of *C. forsteri* ePE and cPE was analyzed on MDMs (Figure 3A) and results revealed no significant increase of cell mortality for ePE (from 14.33 ± 48.58 to 59.83 ± 40.41%) and cPE (from 41.50 ± 110.9 to 89.83 ± 89.20%) treatments compared to LPS-treated cells (103.5 ± 84.77%) and regardless of plant extract concentration. QUIN production was measured, and induction confirmed with LPS with a QUIN concentration at 1,574 ± 482.4 nM. Inhibitory effect on QUIN production (Figures 3B, C) was observed for both ePE (*p*-value = 0.02250) and cPE (*p*-value = 0.01761) extracts compared to LPS condition with significant activity for ePE at 10 μg/ml (QUIN concentration at 671.5 ± 325.5 nM) and cPE at 50 μg/ml (QUIN concentration at 441.2 ± 338.8 nM).

### 4.3 Chemical profile of cyclohexane extract of *C. forsteri* leaf extract

**FIGURE 4:**
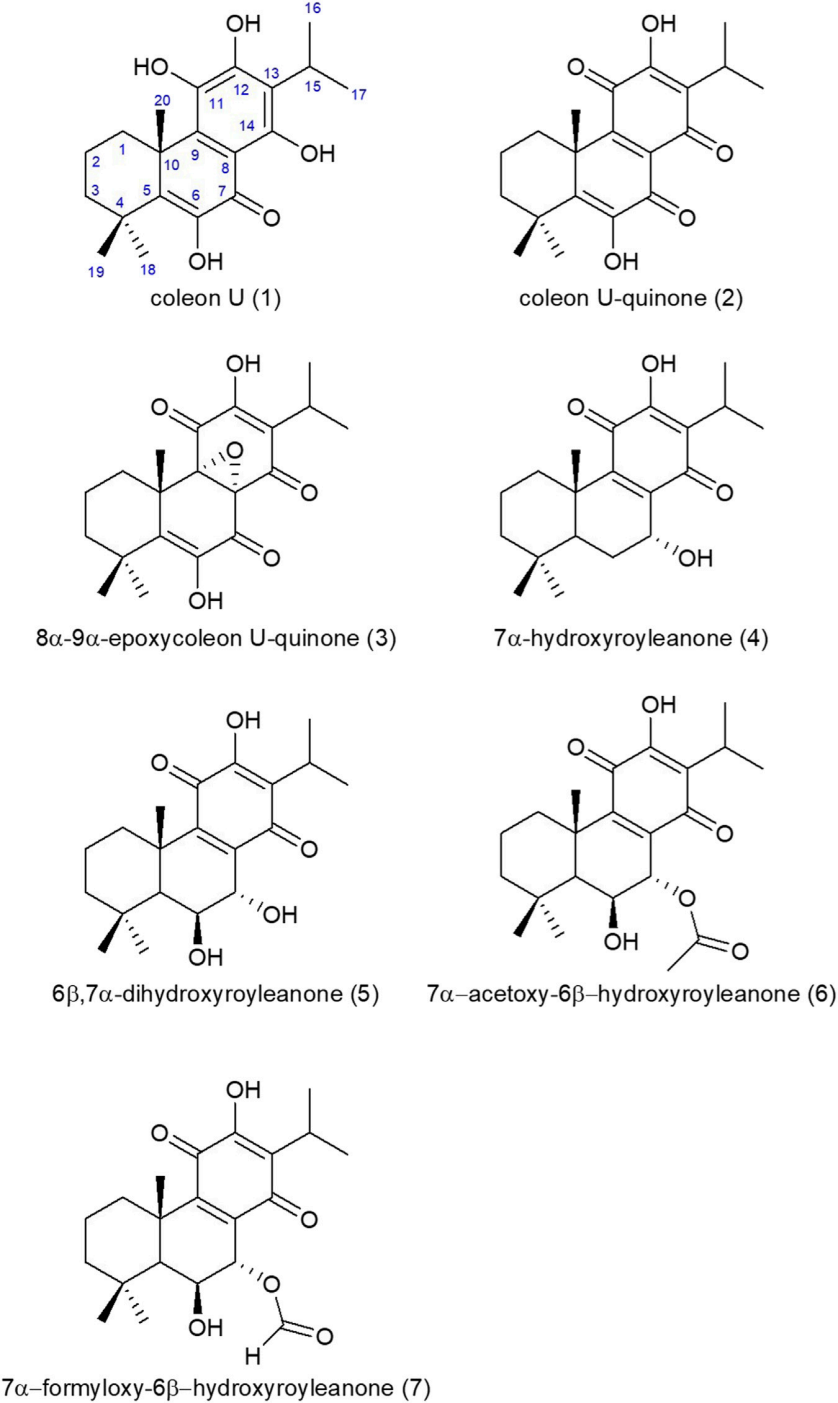
Chemical structures of molecules isolated from *C. forsteri* cyclohexane extract.

Seven abietane diterpenes (Figure 4) were characterized from *C. forsteri* cPE: coleon U (**1**) (Toshio et al., 1977; Wellsow et al., 2006), coleon U-quinone (**2**) (Alder et al., 1984), 8α,9α-epoxycoleon U-quinone (**3**) (Alder et al., 1984), horminone or 7α-hydroxyroyleanone (**4**) (Hensch et al., 1975), 6β,7α-dihydroxyroyleanone (**5**) (Hensch et al., 1975), 7α-acetoxy-6β-hydroxyroyleanone (**6**) (Hensch et al., 1975) and 7α-formyloxy-6β-hydroxyroyleanone (**7**) (Toshio et al., 1977). Purity of isolated compounds was verified by HPLC (see Supplementary Material S1). The compounds (1) to (7) differ from the presence of several polar groups (alcohol, ketone, *etc.,*) brought in evidence by the loss of 18, 28 or 44 m. a.u in mass spectrometry (Table 1). The abietane skeleton is a 20 carbons diterpene consisting in 3 fused 6-membered rings that contains a methyl group at the C10, 2 methyl groups at the C4 and an isopropyl group at the C13 (Supplementary Table S1). These alkyl groups are common to the seven isolated compounds and evidenced by ^1^H NMR (Supplementary Table S2) showing characteristic signals (singlet for methyl, septuplet and two doublets for isopropyl).

## 4 Discussion

*Plectranthus sensu lato* is a large and widespread genus with an important range of ethnobotanical uses including medicinal purposes (Lukhoba et al., 2006; Rice et al., 2011). Phytochemical studies have shown that this genus, including *Coleus* species, is a rich source of diterpenes exerting promising biological activities with abietane diterpenes as the most abundant group (Abdel-Mogib et al., 2002; Gáborová et al., 2022). Interestingly, commercial extract of *C. amboinicus* was shown to inhibit maturation and release of inflammatory cytokine IL-1β through inhibition of nuclear factor NF-kB translocation (Leu et al., 2019). Formerly classified among *Plectranthus* genus, *C. forsteri* is a traditional plant used in the Pacific region especially in New Caledonia, to treat flu-like symptoms and shock-related ecchymosis (Rageau and Schmid, 1973; Suprin, 2008; Lormée et al., 2011). However, only few pharmacological studies have been undertaken on *C. forsteri*. Polyphenols and diterpenoids as well as bioactive caffeic acid esters nepetoidins A and B were found in leaf extract of *C. forsteri* (Grayer et al., 2003; Wellsow et al., 2006; Kubínová et al., 2013). Herein, our results showed that cyclohexane (cPE) and ethanolic (ePE) extracts of *C. forsteri* exert inhibitory activity on the levels of the inflammatory cytokines IL-6 and TNF-α and of the deleterious KP metabolite QUIN produced by human macrophages under inflammatory conditions. Composition of the cyclohexane extract was analyzed and 7 known abietane diterpenes were characterized: coleon U (**1**), coleon U-quinone (**2**), 8α,9α-epoxycoleon U-quinone (**3**), horminone or 7α-hydroxyroyleanone (**4**), 6β,7α-dihydroxyroyleanone (**5**), 7α-acetoxy-6β-hydroxyroyleanone (**6**) and 7α-formyloxy-6β-hydroxyroyleanone (**7**). Previously, nepetoidins A and B were isolated from a methanolic extract of *C. forsteri* while they were not found in cyclohexane extract in our study (Kubínová et al., 2013).

Coleon U (**1**) and coleon U-quinone (**2**) were previously isolated from *C. forsteri* validating our results (Wellsow et al., 2006). Both molecules showed antibacterial activity against phytopathogenic *Pseudomonas syringae* (Wellsow et al., 2006). Coleon U-quinone (**2**) purified from *C. hadiensis* (formerly *P. madagascariensis*) exerted antibacterial against pathogenic *Staphylococcus aureus* and *Enterococcus faecalis* while coleon U (**1**) isolated from *C. grandidentatus* native of Africa, showed interesting antimicrobial activity against methicillin-resistant *S. aureus* (MRSA) and vancomycin-resistant *Enterococcus* (VRE) (Gaspar-Marques et al., 2006; Kubínová et al., 2014). Coleon U (**1**) also showed antifungal activity on *Bacillus subtilis* (Wellsow et al., 2006). Coleon U (**1**) and coleon U-quinone (**2**) also exhibited inhibitory activity on human cancer cell line proliferation (Marques et al., 2002; Matias et al., 2019; Ntungwe et al., 2022). Interestingly, coleon U (**1**) isolated from *C. grandidentatus* also showed antiproliferative activity on human T- and B-cell (Cerqueira et al., 2004).

In this study, compound 8α,9α-epoxycoleon U-quinone (**3**) was newly isolated from *C. forsteri*. It was previously isolated from *C. xanthanthus* and showed cytotoxic activity toward human leukemia cells (Mei et al., 2002). Together with coleon U (**1**) and coleon U-quinone (**2**), 8α,9α-epoxycoleon U-quinone (**3**) was also found in *C. mutabilis* leaves and compounds (**1–3**) were shown to inhibit P-glycoprotein (P-gp) activity in lung cancer cell line (Ntungwe et al., 2022). Related to Multidrug Resistance (MDR), P-gp is a membrane transporter involved in immune response and induced during chronic inflammatory diseases as RA, IBD and SLE (Liu et al., 2015; Wu et al., 2018; Ahmed Juvale et al., 2022). It was shown to induce the release of cytokines from PBMCs treated with anti-inflammatory methotrexate and dexamethasone (Pawlik et al., 2005). Thus, inhibitory action of compounds (**1–3**) on P-gp might contribute to the anti-inflammatory activity of *C. forsteri* through the regulation of inflammatory cytokine production.

Horminone or 7α-hydroxyroyleanone (**4**) is a well-studied royleanone compound isolated from several *Plectranthus s.l.* Species exerting various bioactivities ranging from antitumoral and cytotoxic activities on various cancer cell lines to antimicrobial properties on MRSA and VRE as well as against *Vibrio cholerae* and *Mycobacterium tuberculosis* (Gáborová et al., 2022). Horminone (**4**) inhibitory effect was also studied on KP metabolites and was shown to slightly regulate IDO1 activity and KYN and TRP production (Becker et al., 2018). Thus, horminone (**4**) might contribute to the *C. forsteri* inhibitory activity on QUIN production through the regulation of IDO1 enzymatic activity.

Herein, 6β,7α-dihydroxyroyleanone (**5**) is newly isolated from *C. forsteri* while 7α-acetoxy-6β-hydroxyroyleanone (**6**) was previously isolated from *C. forsteri* methanolic extract (Kubínová et al., 2013). Both compounds exert antimicrobial activity as well as cytotoxicity and inhibitory effect on the growth of human cancer cell lines (Marques et al., 2002; Gaspar-Marques et al., 2006; Matias et al., 2019; Gáborová et al., 2022). It is noticeable that 7α-acetoxy-6β-hydroxyroyleanone (**6**) was shown to yield a potent antiproliferative activity against human lymphocytes with higher efficacy than the immunosuppressor cyclosporin (Cerqueira et al., 2004). Interestingly, compound (**5**) and synthetic royleanone derivatives of (**5**) and (**6**) were shown to inhibit P-gp (Garcia et al., 2020).

The last compound 7α-formyloxy-6β-hydroxyroyleanone (**7**) is newly isolated from *C. forsteri*. It was previously found in other *Coleus* species (Gáborová et al., 2022). However, bioactivities of compound (**7**) were barely studied. No antimicrobial activity was reported and low cytotoxicity on cancer cell line was reported (Matias et al., 2019; Gáborová et al., 2022).

Antibacterial activities of abietane diterpenes (**1**), (**2**), (**4**), (**5**) and (**6**) could participate in biological properties of *C. forsteri* during infection to clear microbial pathogens while anti-inflammatory potential could be related to compounds (**1**), (**3**), (**4**), (**5**) and (**6**) through the regulation KP and/or of P-gp production. Altogether, our results enhance biological properties of *C. forsteri* highlighting the beneficial use of its extracts in the Pacific traditional remedies.

## Data Availability

The original contributions presented in the study are included in the article/Supplementary Material, further inquiries can be directed to the corresponding author.

## Glossary

CD: Crohn’s disease
cPE: cyclohexane plant extract
DMSO: dimethyl sulfoxide
ePE: ethanolic plant extract
EtOH: ethanol
FBS: fetal bovine serum
GC/MS: gas chromatography/mass spectrometry
GM-CSF: granulocyte-macrophage colony-stimulating factor
HEPES: 4-(2-hydroxyethyl)-1-piperazineethanesulfonic acid
IBD: inflammatory bowel disease
IDO1: 2,3-dioxygenase
IFNs: interferons
ILs: interleukins
IMIDs: immune-mediated inflammatory diseases
IPNC: Institut Pasteur of New Caledonia
KP: kynurenine pathway
KYN: kynurenine
LDH: lactate dehydrogenase
LPS: lipopolysaccharide
MDMs: monocyte-derived macrophages
MRSA: methicillin-resistant *Staphylococcus aureus*
MS: mass spectrometry
NSAIDs: non-steroidal anti-inflammatory drugs
PBMCs: peripheral blood mononuclear cells
P-gp: P-glycoprotein
PMA: phorbol 12-myristate 13-acetate
QUIN: quinolinic acid
RA: rheumatoid arthritis
RPMI: Roswell Park Memorial Institute
SD: standard deviation
SLE: systemic lupus erythematosus
SpA: spondyloarthritis
TNF: tumor necrosis factor
TRP: tryptophan
UC: ulcerative colitis
UNC: University of New Caledonia
VRE: vancomycin-resistant *Enterococcus*
