# Supplementary material for: Anti-inflammatory activities of Coleus forsteri (formerly Plectranthus forsteri) extracts on human macrophages and chemical characterization
Source: Front Pharmacol. 2023 Jan 9;13:1081310. doi: 10.3389/fphar.2022.1081310 (PMC9868419; doi:10.3389/fphar.2022.1081310)
Supplement: Supplementary file 1 [file Presentation1.ppt]

## Slide 1
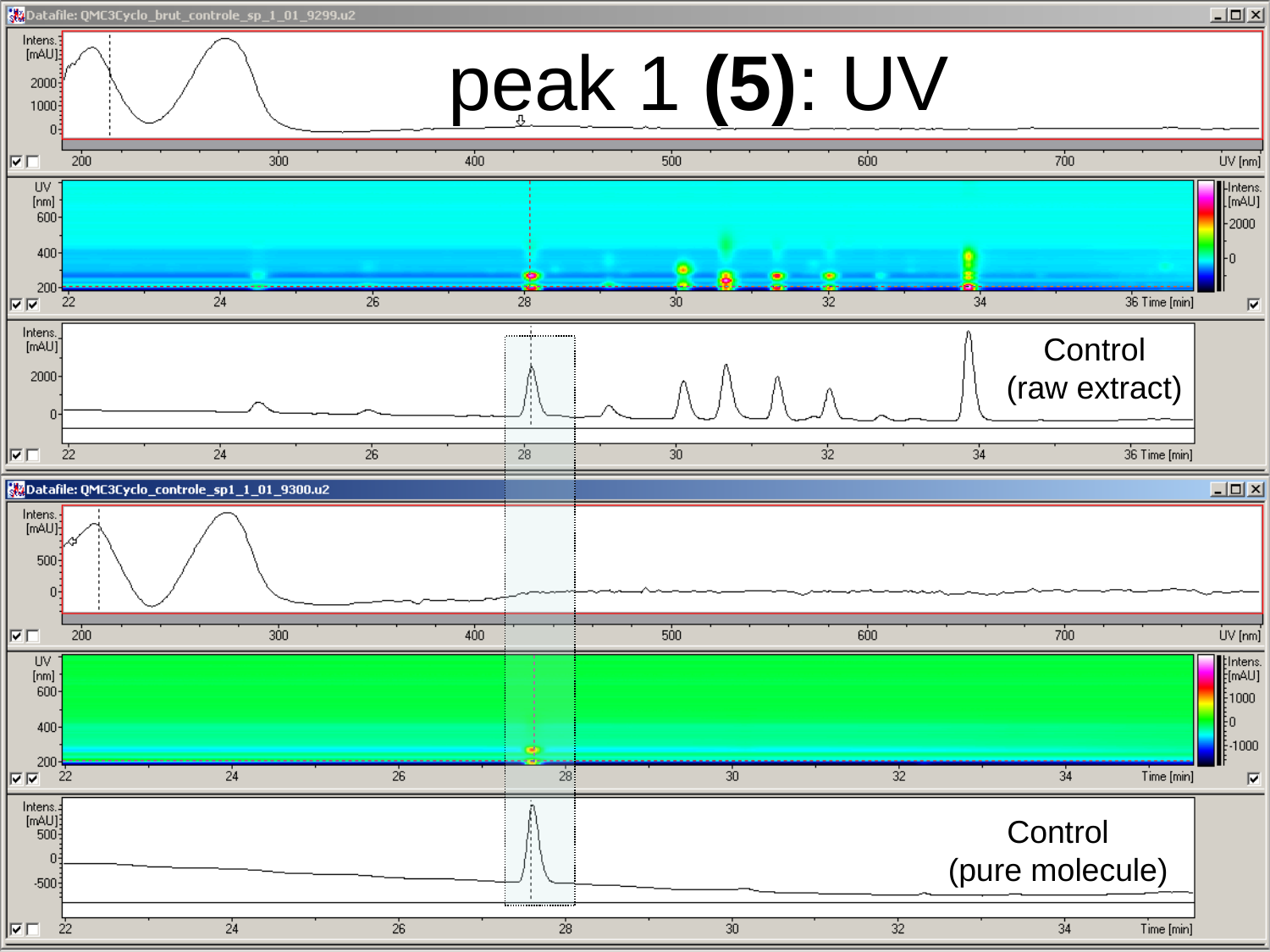

peak 1 (5): UV
Control
(raw extract)
# peak 1
Control
(pure molecule)

## Slide 2
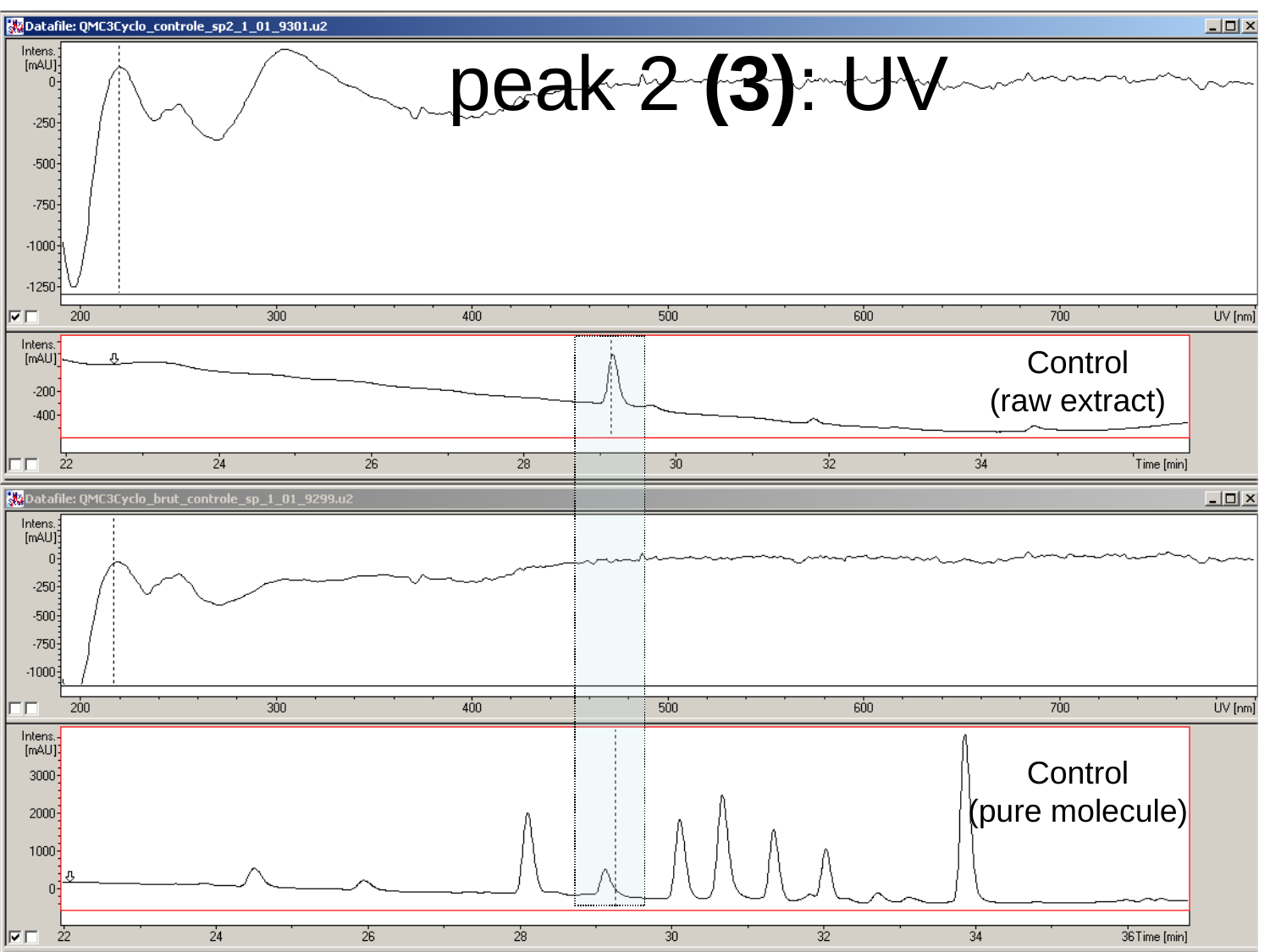

peak 2 (3): UV
Control
(raw extract)
Control
(pure molecule)

## Slide 3
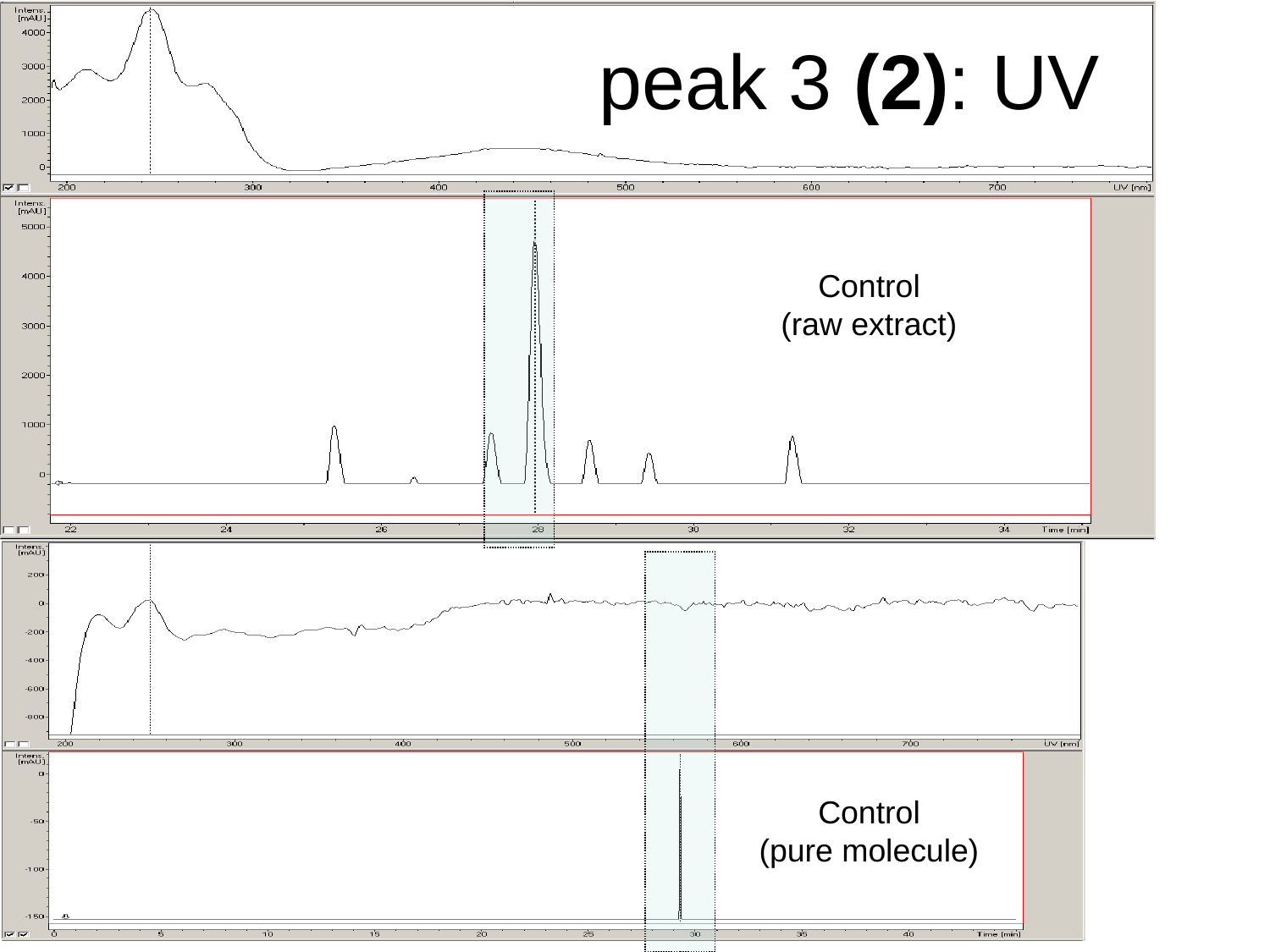

peak 3 (2): UV
Control
(raw extract)
Control
(pure molecule)

## Slide 4
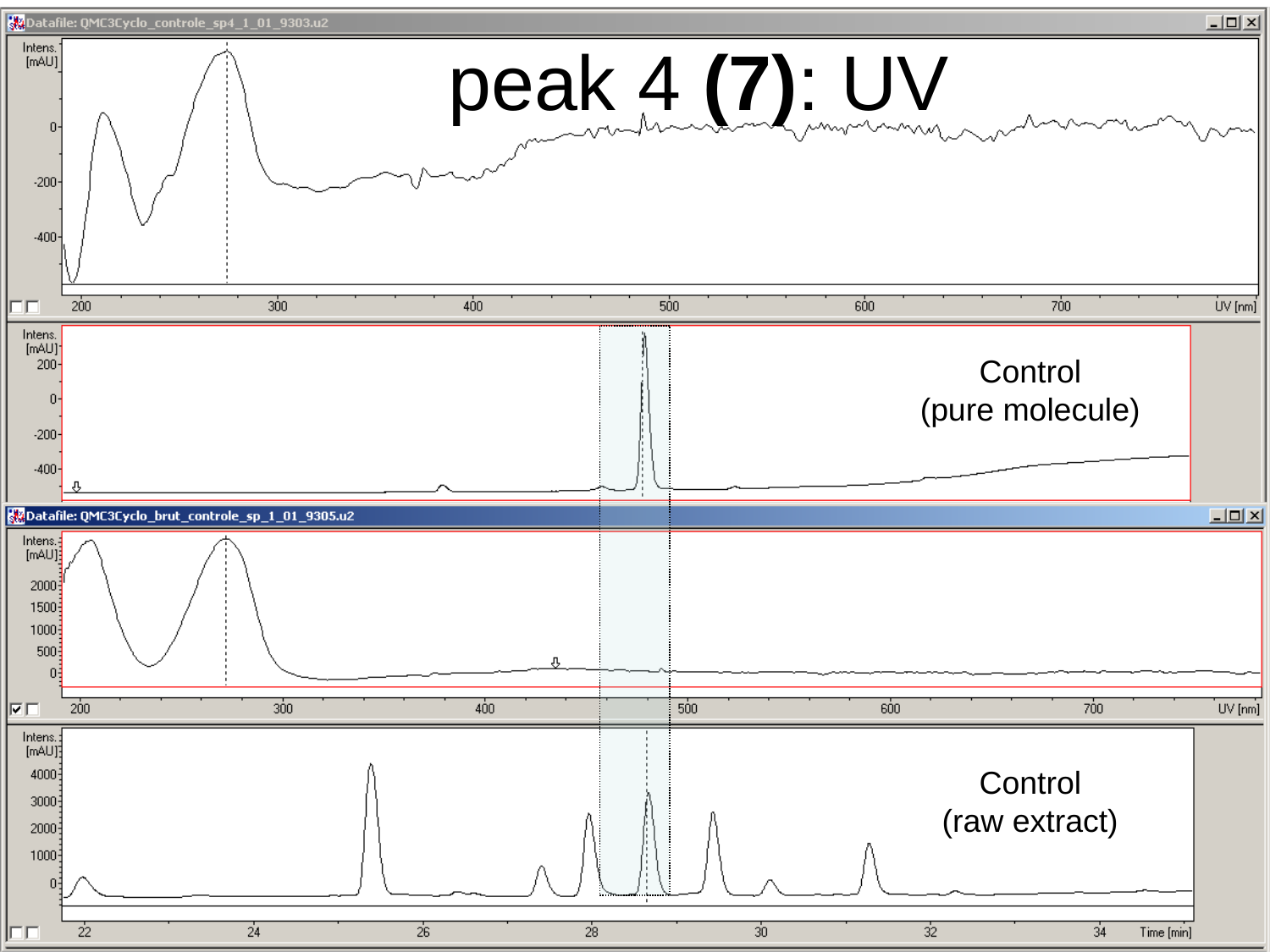

peak 4 (7): UV
#
Control
(pure molecule)
Control
(raw extract)

## Slide 5
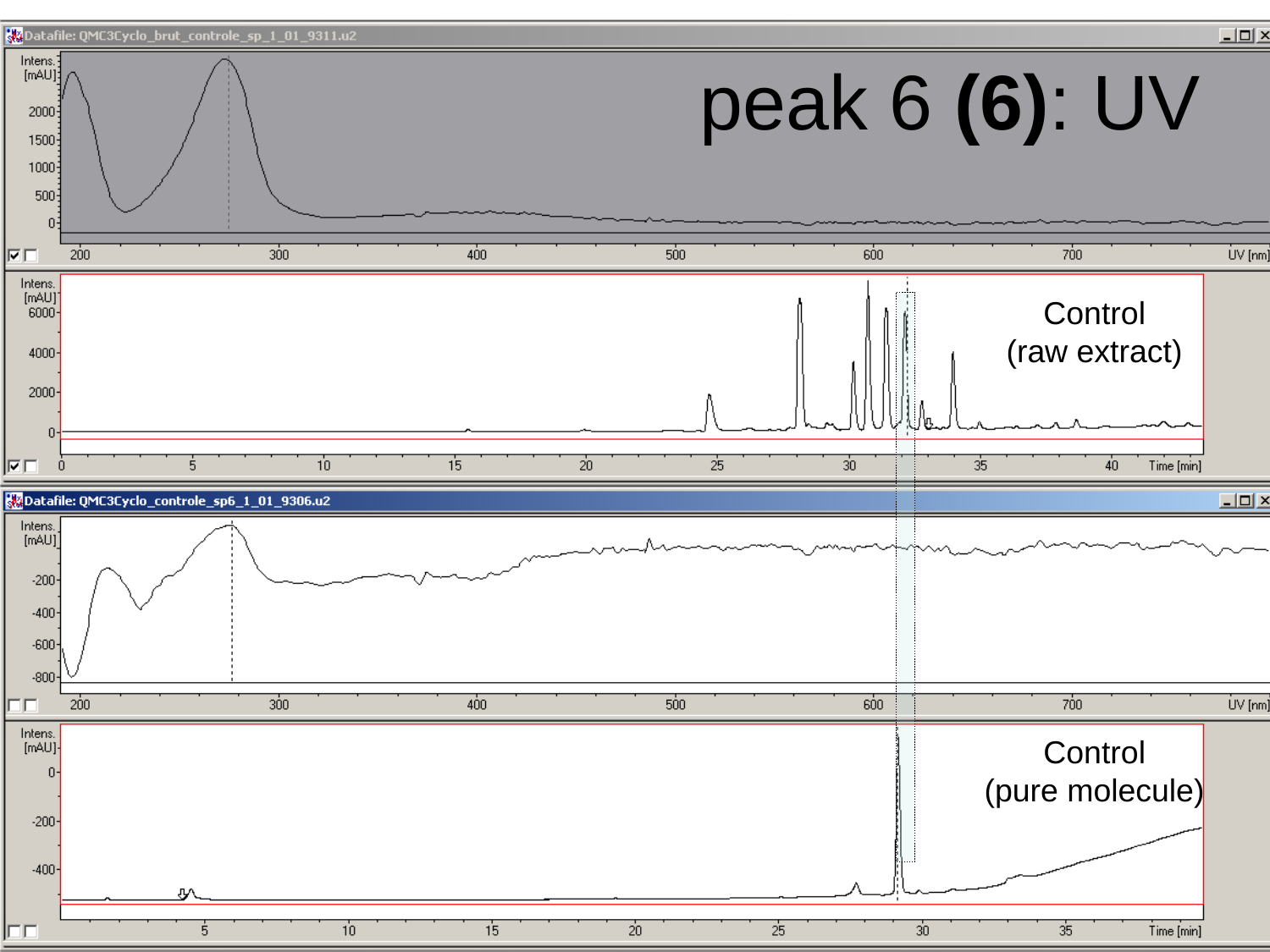

peak 6 (6): UV
#
Control
(raw extract)
Control
(pure molecule)

## Slide 6
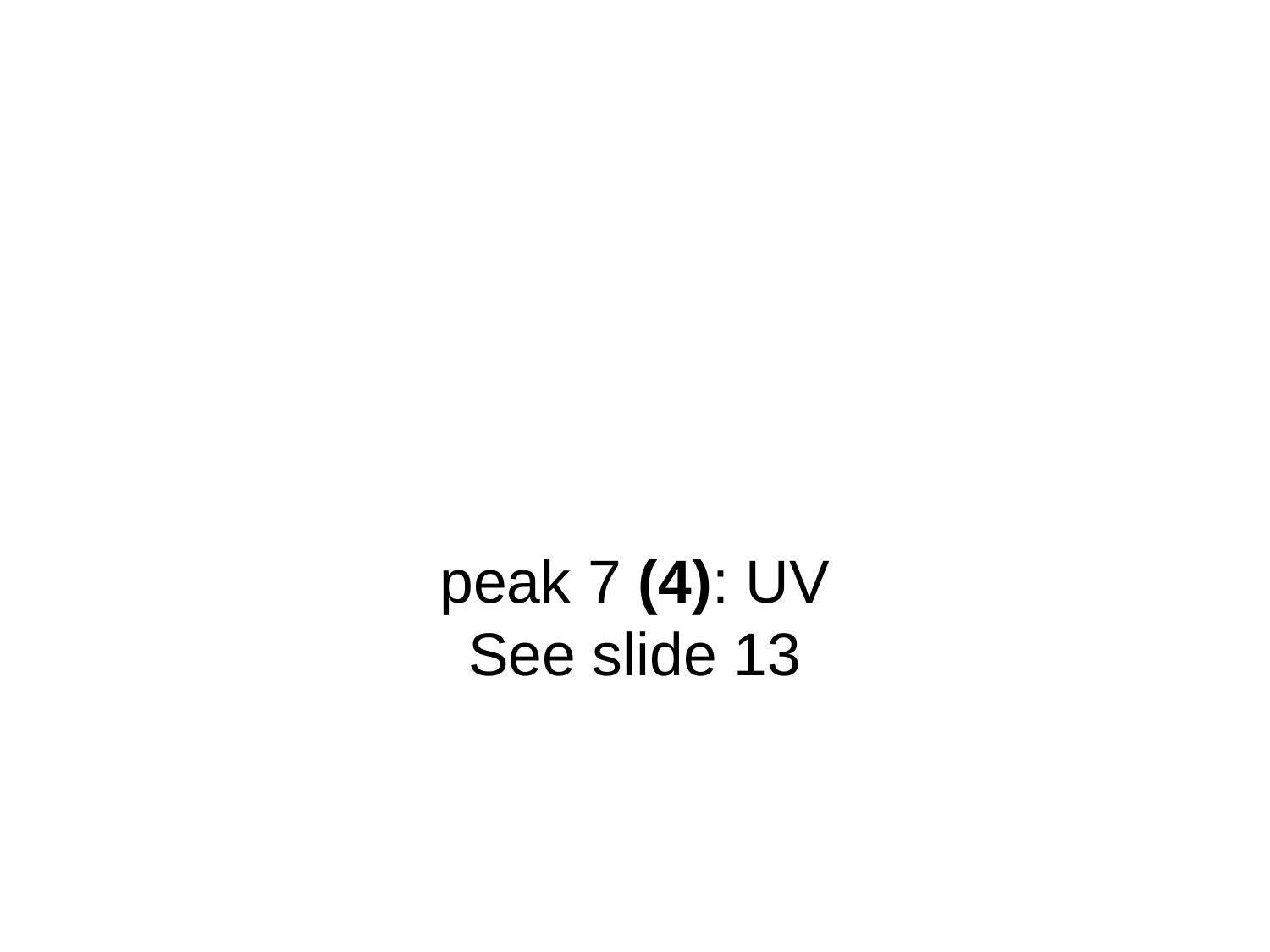

peak 7 (4): UV
See slide 13

## Slide 7
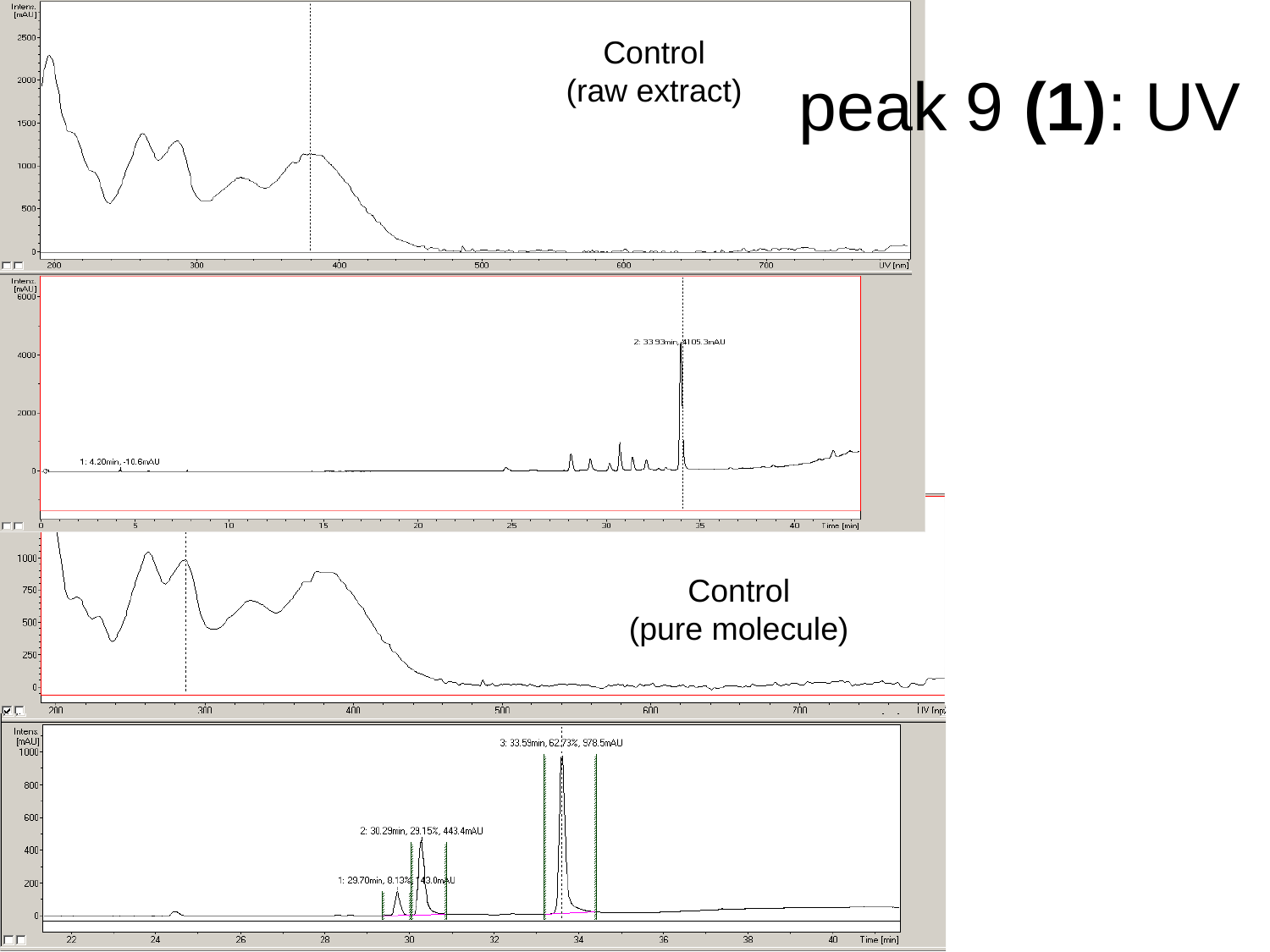

peak 9 (1): UV
Control
(raw extract)
Control
(pure molecule)

## Slide 8
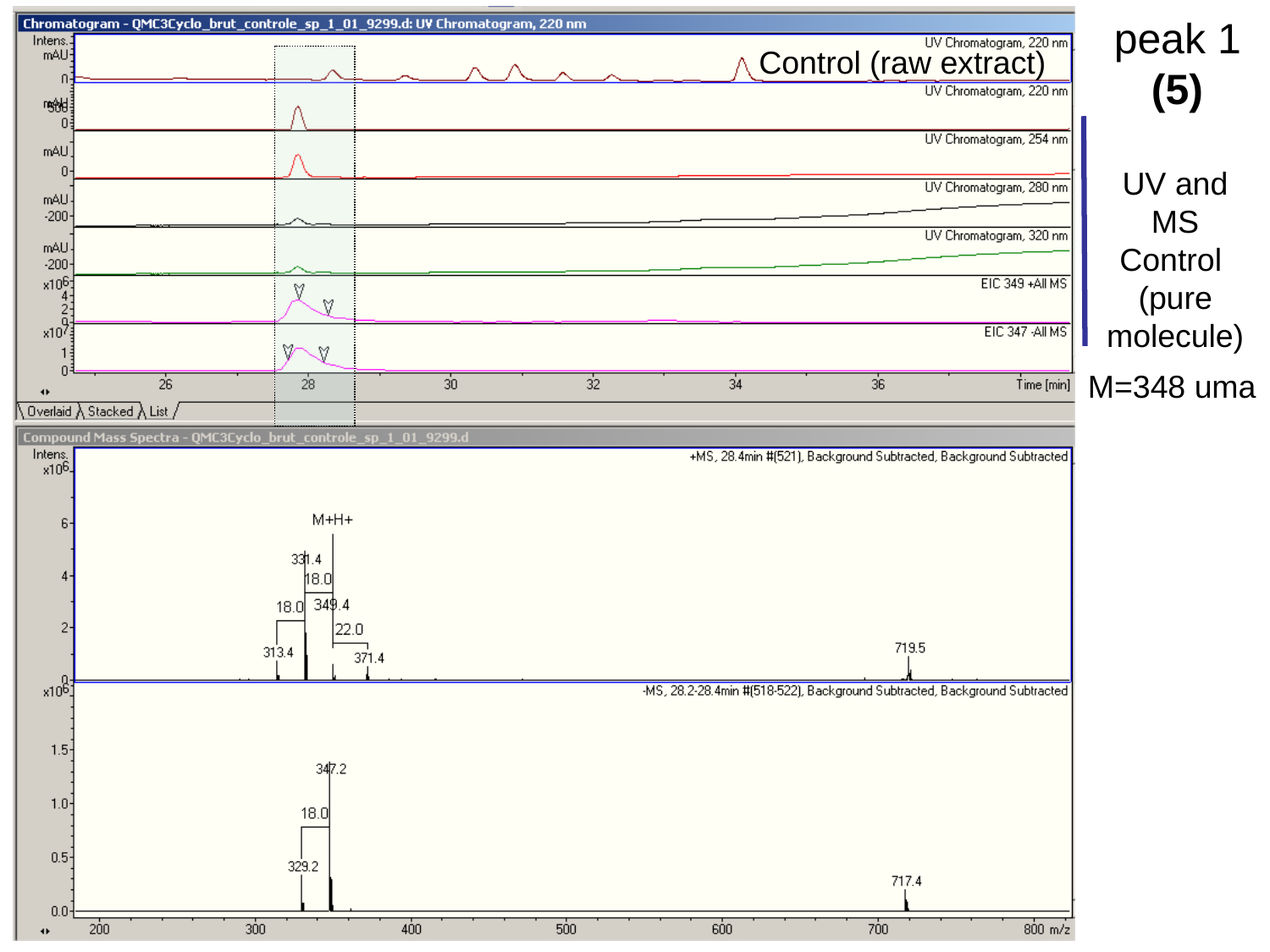

# peak 1 (5)
Control (raw extract)
UV and MS Control
(pure molecule)
M=348 uma

## Slide 9
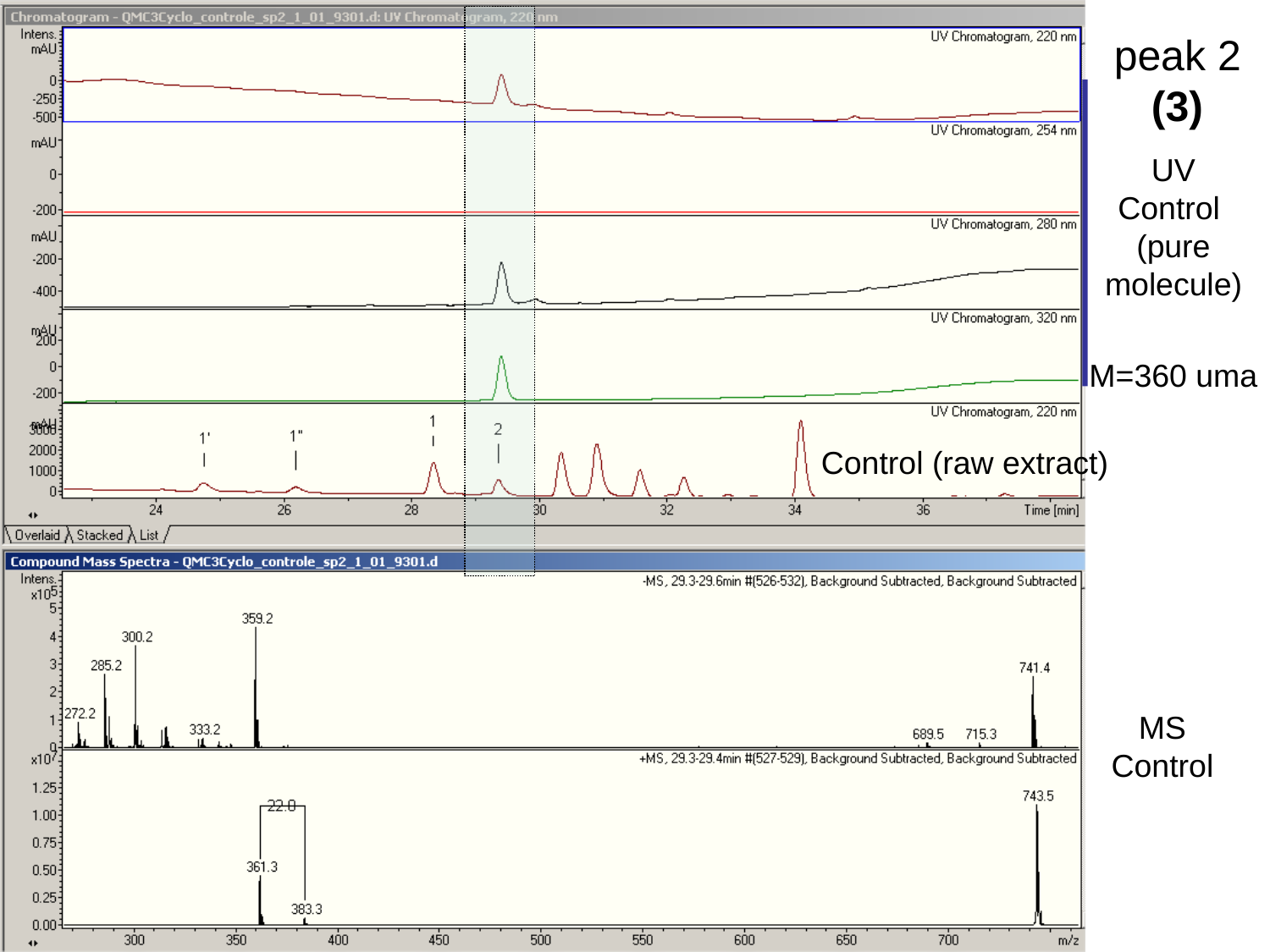

# peak 2 (3)
UV Control
(pure molecule)
M=360 uma
Control (raw extract)
MS
Control

## Slide 10
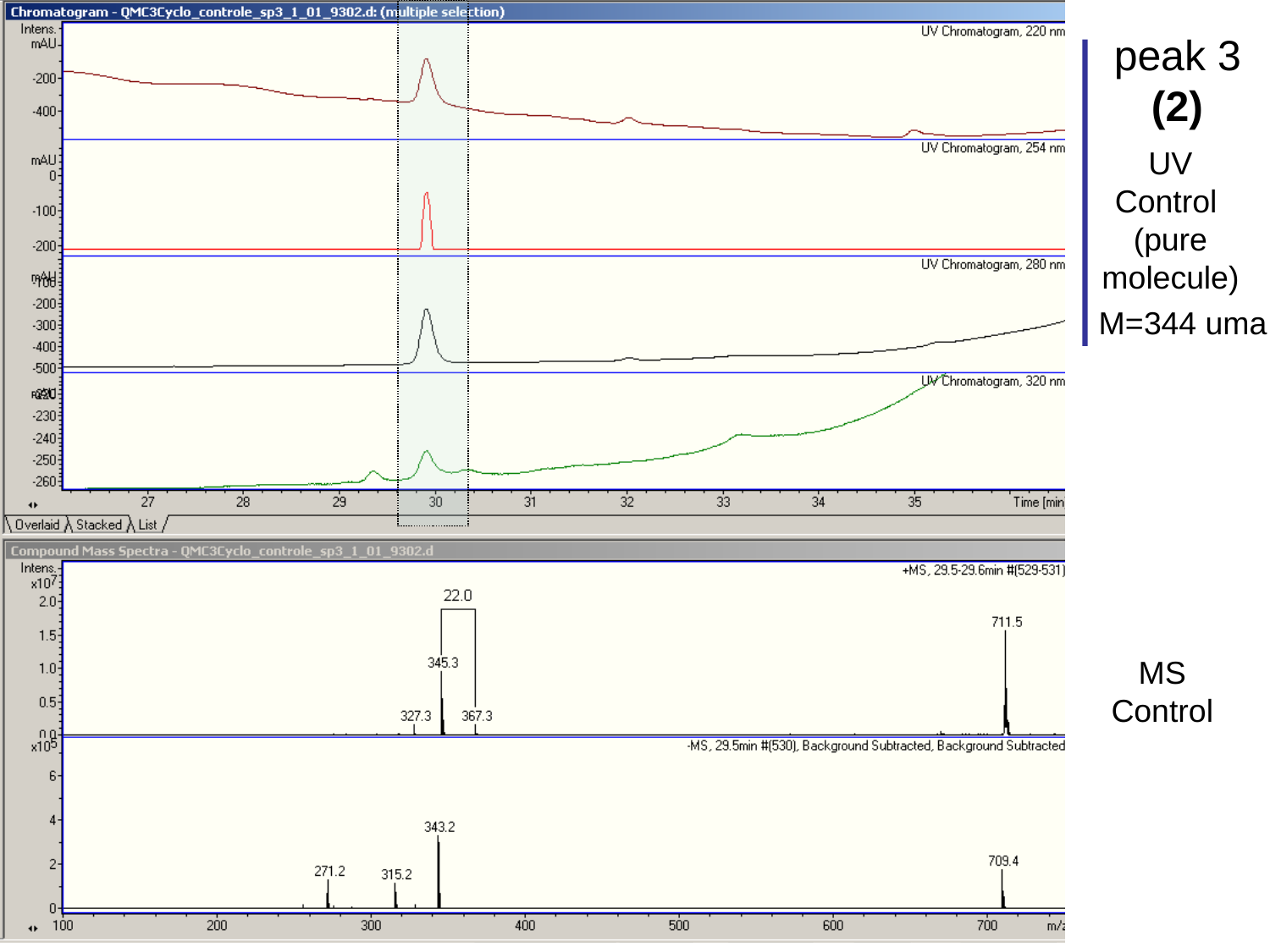

# peak 3 (2)
UV Control
(pure molecule)
M=344 uma
MS
Control

## Slide 11
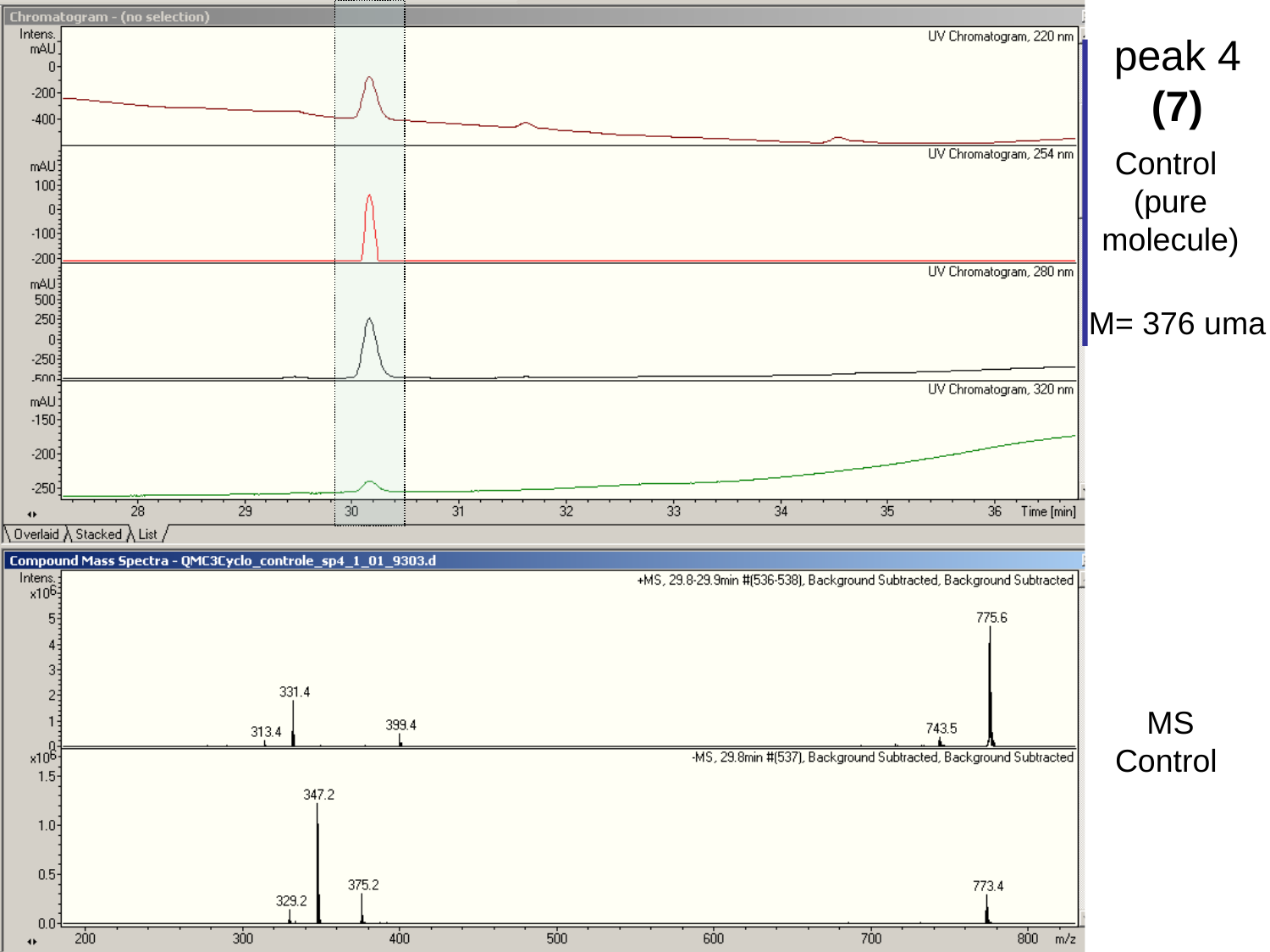

# peak 4 (7)
Control
(pure molecule)
M= 376 uma
MS
Control

## Slide 12
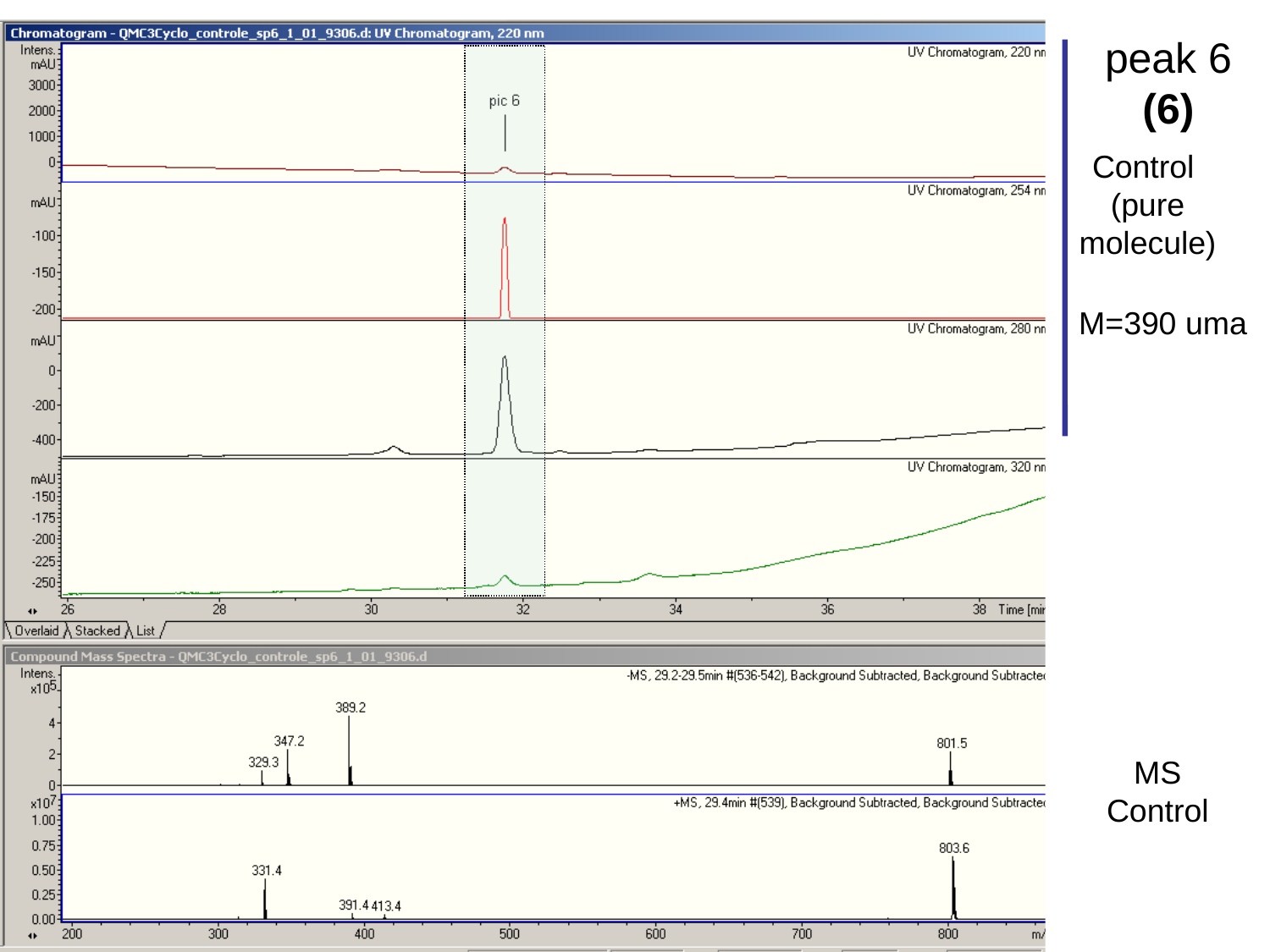

# peak 6 (6)
Control
(pure molecule)
M=390 uma
MS
Control

## Slide 13
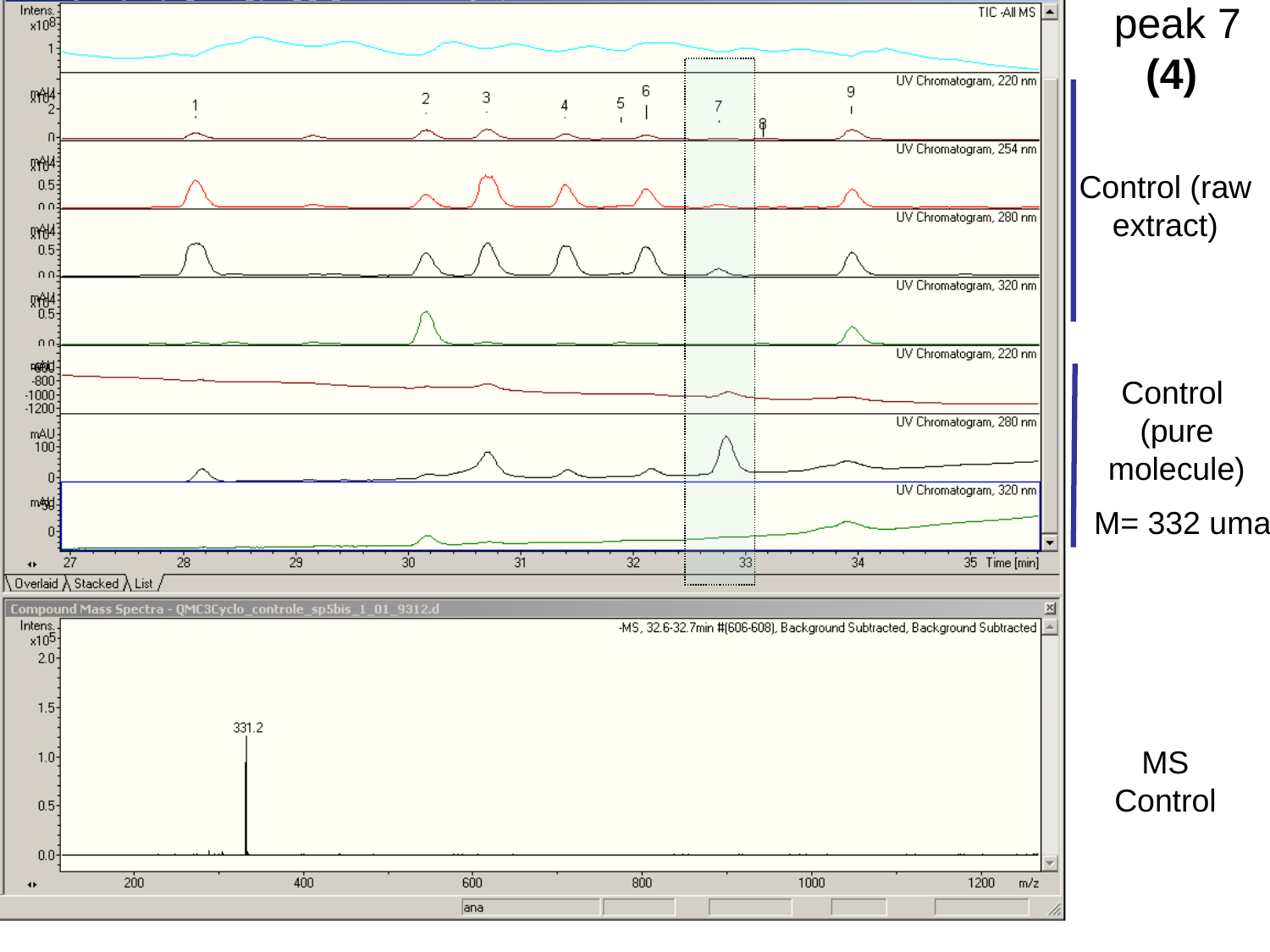

# peak 7 (4)
Control (raw extract)
Control
(pure molecule)
M= 332 uma
MS
Control

## Slide 14
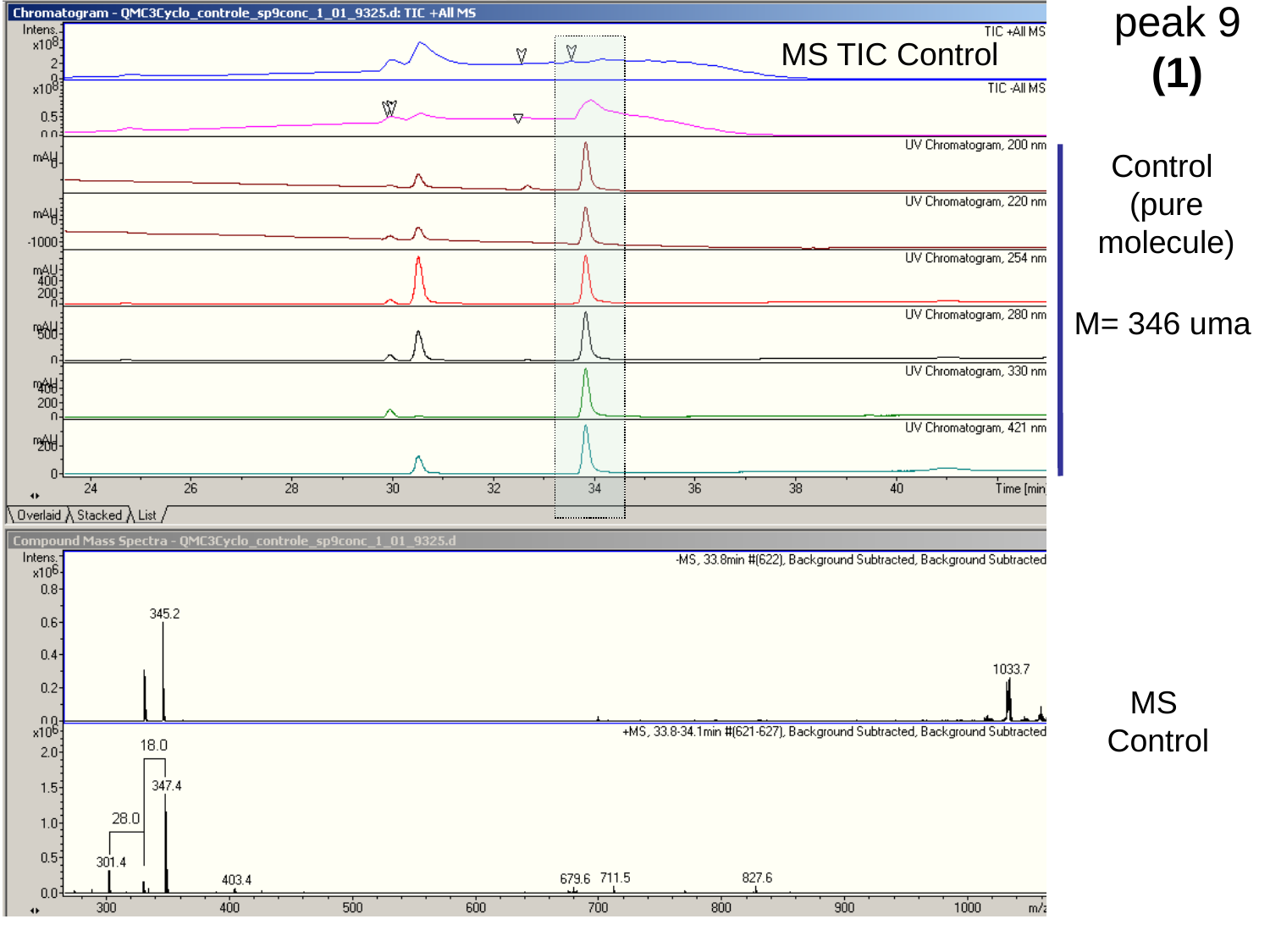

# peak 9 (1)
MS TIC Control
Control
(pure molecule)
M= 346 uma
MS
Control
