# Supplementary material for: Anti-inflammatory activities of Coleus forsteri (formerly Plectranthus forsteri) extracts on human macrophages and chemical characterization
Source: Front Pharmacol. 2023 Jan 9;13:1081310. doi: 10.3389/fphar.2022.1081310 (PMC9868419; doi:10.3389/fphar.2022.1081310)
Supplement: Supplementary file 2 [file Table1.docx]

Anti-inflammatory Activities of *Coleus forsteri* (formerly *Plectranthus forsteri*) Extracts on Human Macrophages and Chemical Characterization

Mael NICOLAS^1±†^, Malia LASALO^2±^, Sharron CHOW^3^, Cyril ANTHEAUME^4†^, Karl HUET^2^, Edouard HNAWIA^5^, Gilles J. GUILLEMIN^3#^, Mohammed NOUR^6#^, Mariko MATSUI^2,6#*^

**Suppl. Table 1: RMN ^13^C data for compounds (1) to (7) isolated from *C. forsteri* cyclohexane extract.**

|  | **Compounds** | | | | | | |
| --- | --- | --- | --- | --- | --- | --- | --- |
| C atoms | **1** | **2** | **3** | **4** | **5** | **6** | **7** |
| 1 | 31.7 | 30.9 | 31.2 | 35.9 | 38.5 | 38.5 | 38.4 |
| 2 | 18.9 | 17.8 | 17.2 | 19.0 | 19.2 | 19.1 | 19.1 |
| 3 | 37.6 | 36.4 | 37.2 | 41.2 | 42.4 | 42.4 | 42.4 |
| 4 | 37.5 | 36.5 | 36.1 | 33.2 | 33.9 | 33.8 | 33.8 |
| 5 | 144.1 | 143.7 | 140.1 | 45.9 | 49.6 | 49.9 | 49.8 |
| 6 | 143.3 | 146.9 | 140.9 | 25.9 | 69.4 | 67.2 | 67.2 |
| 7 | 184.5 | 177.7 | 183.0 | 63.3 | 69.3 | 68.9 | 68.3 |
| 8 | 106.2 | 126.9 | 56.8 | 143.3 | 141.1 | 137.2 | 136.4 |
| 9 | 140.7 | 155.3 | 67.7 | 148.0 | 147.6 | 150.0 | 150.4 |
| 10 | 42.5 | 41.6 | 36.1 | 39.2 | 38.7 | 38.7 | 38.8 |
| 11 | 135.6 | 183.6 | 186.6 | 184.0 | 183.5 | 183.4 | 183.3 |
| 12 | 157.8 | 150.9 | 150.8 | 151.2 | 151.3 | 151.0 | 151.1 |
| 13 | 120.9 | 126.1 | 128.5 | 124.3 | 124.4 | 124.8 | 124.9 |
| 14 | 158.2 | 184.6 | 186.4 | 189.3 | 189.3 | 185.9 | 185.8 |
| 15 | 25.8 | 24.5 | 25.5 | 24.1 | 24.1 | 24.3 | 24.3 |
| 16 | 20.6 | 19.9 | 19.7 | 20.0 | 19.9 | 20.0 | 20.0 |
| 17 | 20.6 | 19.9 | 19.3 | 19.9 | 19.9 | 19.8 | 19.8 |
| 18 | 27.6 | 27.6 | 28.4 | 33.3 | 33.7 | 33.7 | 33.6 |
| 19 | 28.5 | 27.2 | 27.2 | 21.8 | 24.4 | 24.0 | 24.0 |
| 20 | 28.7 | 29.2 | 27.5 | 18.5 | 21.7 | 21.6 | 21.7 |
| 7-O_2_CH | - | - | - | - | - | - | 159.7 |
| 7-OCOCH_3_ | - | - | - | - | - | 169.8 | - |
| 7-OCOCH_3_ | - | - | - | - | - | 21.1 | - |
