# Supplementary material for: Anti-inflammatory activities of Coleus forsteri (formerly Plectranthus forsteri) extracts on human macrophages and chemical characterization
Source: Front Pharmacol. 2023 Jan 9;13:1081310. doi: 10.3389/fphar.2022.1081310 (PMC9868419; doi:10.3389/fphar.2022.1081310)
Supplement: Supplementary file 3 [file Table2.docx]

Anti-inflammatory Activities of *Coleus forsteri* (formerly *Plectranthus forsteri*) Extracts on Human Macrophages and Chemical Characterization

Mael NICOLAS^1±†^, Malia LASALO^2±^, Sharron CHOW^3^, Cyril ANTHEAUME^4†^, Karl HUET^2^, Edouard HNAWIA^5^, Gilles J. GUILLEMIN^3#^, Mohammed NOUR^6#^, Mariko MATSUI^2,6#*^

**Suppl. Table 2: RMN ^1^H data for compounds (1) to (7) isolated from *C. forsteri* cyclohexane extract.**

|  | **Compounds** | | | | | | |
| --- | --- | --- | --- | --- | --- | --- | --- |
| **C atoms** | **1** | **2** | **3** | **4** | **5** | **6** | **7** |
| 1 | 3.15 (m, 1H)  1.67 (m, 1H) | 2.65 (m, 1H)  1.60 (m, 1H) | 2.78 (m, 1H)  1.84 (m, 1H) | 2.69 (dtdl, 13.1, 3.3, 1.3, 1H)  1.18 (tdl, 13.1, 3.8, 1H) | 2.59 (dtl, 12.7, 3.3, 1H)  1.18 (tdl, 13.3, 3.8, 1H) | 2.63 (dtl, 12.7, 3.6, 1H)  1.20 (ov, 1H) | 2.63 (dtl, 13.1, 3.5, 1H)  1.20 (ov, 1H) |
| 2 | 1.87 (m, 1H)  1.70 (m, 1H) | 1.90 (m, 1H)  1.60 (m, 1H) | 1.86 (m, 1H)  1.67 (m, 1H) | 1.73 (qtl, 13.8, 3.6, 1H)  1.57 (m, 1H) | 1.83 (qtl, 13.6, 3.4, 1H)  1.57 (dquinl, 14.1, 3.6, 1H) | 1.85 (qtl, 13.7, 3.6, 1H)  1.57 (dquinl, 13.9, 3.6, 1H) | 1.85 (qtl, 13.9, 3.6, 1H)  1.57 (dquinl, 13.9, 3.6, 1H) |
| 3 | 2.04 (tdl, 12.4, 5.3, 1H)  1.40 (m, 1H) | 1.99 (tdl, 13.1, 4.7, 1H)  1.47 (m, 1H) | 1.80 (m, 1H)  1.40 (m, 1H) | 1.47 (dtdl, 13.8, 3.6, 1.2, 1H)  1.24 (ov, 1H) | 1.47 (dtdl, 13.4, 3.3, 1.0, 1H)  1.26 (tdl, 13.7, 3.4, 1H) | 1.48 (dtl, 13.4, 3.6, 1H)  1.22 (ov, 1H) | 1.48 (dtl, 13.6, 3.6, 1H)  1.22 (ov, 1H) |
| 4 | - | - | - | - | - | - | - |
| 5 | - | - | - | 1.52 (ddl, 13.1, 1.1, 1H) | 1.44 (sl, 1H) | 1.34, (sl, 1H) | 1.37, (sl, 1H) |
| 6 | - | - | - | 1.96 (dl, 14., 1H)  1.61 (tdl, 13.3, 4.7, 1H) | 4.45 (sl, 1H) | 4,31 (sl, 1H) | 4,36 (sl, 1H) |
| 7 | - | - | - | 4.72 (dl, 4.7, 1H) | 4.51 (sl, 1H) | 5,65 (dl, 2.0, 1H) | 5,80 (sl, 1H) |
| 8 | - | - | - | - | - | - | - |
| 9 | - | - | - | - | - | - | - |
| 10 | - | - | - | - | - | - | - |
| 11 | - | - | - | - | - | - | - |
| 12 | - | - | - | - | - | - | - |
| 13 | - | - | - | - | - | - | - |
| 14 | - | - | - | - | - | - | - |
| 15 | 3.47 (spt, 7.1, 1H) | 3.22 (spt, 7.1, 1H) | 3.14 (spt, 7.1, 1H) | 3.15 (spt, 7.1, 1H) | 3.16 (spt, 7.1, 1H) | 3.16 (spt, 7.1, 1H) | 3.16 (spt, 7.1, 1H) |
| 16 | 1.33 (d, 7.1, 3H) | 1.25 (d, 7.1, 3H) | 1.23 (d, 7.1, 3H) | 1.21 (d, 7.1, 3H) | 1.22 (d, 7.1, 3H) | 1.23 (d, 7.1, 3H) | 1.22 (d, 7.1, 3H) |
| 17 | 1.33 (d, 7.1, 3H) | 1.25 (d, 7.1, 3H) | 1.20 (d, 7.1, 3H) | 1.20 (d, 7.1, 3H) | 1.22 (d, 7.1, 3H) | 1.19 (d, 7.1, 3H) | 1.19 (d, 7.1, 3H) |
| 18 | 1.46 (s, 3H) | 1.42 (s, 3H) | 1.36 (s, 3H) | 0.98 (s, 3H) | 1.04 (s, 3H) | 0.94 (s, 3H) | 0.96 (s, 3H) |
| 19 | 1.43 (s, 3H) | 1.41 (s, 3H) | 1.35 (s, 3H) | 0.90 (s, 3H) | 1.25 (s, 3H) | 1.22 (s, 3H) | 1.23 (s, 3H) |
| 20 | 1.66 (s, 3H) | 1.64 (s, 3H) | 1.54 (s, 3H) | 1.21 (s, 3H) | 1.60 (s, 3H) | 1.61 (s, 3H) | 1.60 (s, 3H) |
| 6-OH | - | 7.09 (sl, 1H) | 6.34 (s, 1H) | - | 3.01 (sl, 1H) | 1.97 (d, 3.6, 1H) | 1.97 (d, 3.9, 1H) |
| 7-OH | - | - | - | 3.01 (sl, 1H) | 1.52 (sl, 1H) | - | - |
| 7-OCOCH_3_ | - | - | - | - | - | 2.05(s, 3H) | - |
| 7-OCHO | - | - | - | - | - | - | 8.05 (d, 1.0, 1H) |
| 12-OH | - | 7.10 (sl, 1H) | 6.98 (sl, 1H) | 7.23 (sl, 1H) | 7.31 (sl, 1H) | 7.21 (s, 1H) | 7.22 (s, 1H) |
